# Antimicrobial and antioxidant activities of *Streptomyces* species from soils of three different cold sites in the Fez-Meknes region Morocco

**DOI:** 10.1038/s41598-022-21644-z

**Published:** 2022-10-14

**Authors:** Said Rammali, Lahoucine Hilali, Khadija Dari, Bouchaib Bencharki, Abdellatif Rahim, Mohammed Timinouni, Fatima Gaboune, Mohamed El Aalaoui, Abdelkrim khattabi

**Affiliations:** 1 Agri-food and Health Laboratory, Faculty of Sciences and Techniques, Hassan First University of Settat, Km 3, B.P. : 577 Route de Casablanca, 26000 Settat, Morocco; 2 Laboratory of Biochemistry, Neurosciences, Natural Ressources and Environment, Faculty of Sciences and Techniques, Hassan First University of Settat, Km 3, B.P. : 577 Route de Casablanca, 26000 Settat, Morocco Settat; 3 Molecular Bacteriology Laboratory, Institut Pasteur du Maroc, Casablanca, Morocco Place Louis Pasteur, 20100; 4 Biotechnology Unit, National Institute of Agronomic Research of Rabat, Rabat, Morocco Av. Annasr, Rabat 10000; 5 Department of plant protection, Regional Center of Agronomic Research of Settat, Tertiary Road 1406, At 5 Km from Settat, 26400 Settat, Morocco Settat

**Keywords:** Biochemistry, Immunology, Microbiology

## Abstract

The increasing demand for new bioactive compounds to combat the evolution of multi-drug resistance (MDR) requires research on microorganisms in different environments in order to identify new potent molecules. In this study, initial screening regarding the antimicrobial activity of 44 *Actinomycetes* isolates isolated from three soil samples from three different extremely cold sites in Morocco was carried out. Primary and secondary screening were performed against *Candida albicans* ATCC 60,193, *Escherichia coli* ATCC 25,922, *Staphylococcus aureus* ATCC 25,923, *Bacillus cereus* ATCC 14,579, other clinical MDR bacteria, and thirteen phytopathogenic fungi. Based on the results obtained, 11 active isolates were selected for further study. The 11microbial isolates were identified based on morphological and biochemical characters and their molecular identification was performed using 16S rRNA sequence homology. The UV–visible analysis of dichloromethane extracts of the five *Streptomyces* sp. Strains that showed high antimicrobial and antioxidant (ABTS 35.8% and DPPH 25.6%) activities revealed the absence of polyene molecules. GC–MS analysis of the dichloromethane extract of E23-4 as the most active strain revealed the presence of 21 volatile compounds including Pyrrolopyrazine (98%) and Benzeneacetic acid (90%). In conclusion, we studied the isolation of new *Streptomyces* strains to produce new compounds with antimicrobial and antioxidant activities in a cold and microbiologically unexplored region of Morocco. Furthermore, this study has demonstrated a significant (*P* < 0.0001) positive correlation between total phenolic and flavonoid contents and antioxidant capacity, paving the way for the further characterization of these *Streptomyces* sp. isolates for their optimal use for anticancer, antioxidant, and antimicrobial purposes.

## Introduction

Emergent multi-drug resistant (MDR) bacteria including the ESKAPE pathogens (*Enterococcus faecium*, *Staphylococcus aureus*, *Klebsiella pneumoniae*, *Acinetobacter baumannii*, *Pseudomonas aeruginosa*, and *Enterobacter*) represent a global threat to human health and may lead to severe infections that are difficult to treat, resulting in prolonged illness and over times, increased risk of death^1^. Imbalance between the synthesis and accumulation of reactive oxygen species (ROS) in cells and tissues has been shown to cause oxidative stresses that can lead to cancer, cardiovascular, kidney, and neurological diseases^2^. In this context, many previous studies have been oriented towards the search for microorganisms in different environments in order to identify new potent molecules with a wide spectrum of activities^3^. Recently, natural molecules of microbial origin have become the main sources for the production of constantly used antibiotics^4,5^. In addition, it has been estimated that 70% of secondary metabolites are derived from microbial sources^3,6^. Soil contains many ecological niches, and their microbes produce various bio-molecules that are biologically active against a wide range of pathogens^7^. Also, more than 95% of the *Actinomycetes* strains were isolated from soil and *Streptomyces* sp*.* represent the dominant genus^8^. *Streptomyces* belonging to the *Actinomyceaceae* family and are recognized as potential producers of secondary metabolites, including antibiotic, anti-cancer, antioxidant, antimicrobial, and immunosuppressant agents^9^. *Streptomyces* are Gram-positive, filamentous, spore-forming bacteria that have a relatively large genomic (DNA) sequence with a high guanine and cytosine (G + C) content^1,10^. *Streptomyces* comprise a very important bacterial genus that contains over 800 species^11^ which are recognized worldwide as an inexhaustible source of antibiotics used in human therapy^3,12,13^. Antibiotics derived from the *Streptomyces* genus play a crucial role in the medical sector. In addition, it has been highlighted that this genus produces around 50% of therapeutically useful antibiotics^14^. It has also been shown that each *Streptomyces* strain has the capacity to produce more than 30 secondary metaboliteson average^15^.

The majority of *Streptomyces* are aerobic. However, when conditions are limited, *Streptomyces* produce aerial hyphae that can divide to produce spores which can withstand unfavorable conditions and are easily transferred to other environments and nutrient sources^1^. During this phase, *Streptomyces* produce some highly versatile natural products (NPs) that are not required for growth or/and reproduction, but help protect their host against pathogens^1^.

To adapt and survive in extremely cold or cryogenic environments, some microorganisms including *Streptomyces* can produce secondary metabolites of great interest for biotechnological applications^16^. Considering this, we oriented our research towards new bio-active molecules with a wide spectrum of activities extracted from *Streptomyces* sp. isolated in extremely cold environments. To the best of our knowledge, there is no information available regarding the biological and antimicrobial activities of *Streptomyces* strains isolated from extremely cold environments in Morocco. Therefore, the objectives of the present study were to: (1) isolate *Streptomyces* species from the soils of three different cold sites in the Fez-Meknes region, Morocco (Sebt Jahjouh El Hajeb, Ain Vittel Ifrane forest, and Azrou forest); (2) conduct a primary screening of *Streptomyces* isolates for their antimicrobial activity; (3) perform a morphological, biochemical, and physiological characterization, and molecular identification by 16 s rRNA gene sequencing of the 11isolates that showed the highest antimicrobial activity (non-pathogenic bacteria and fungi); (4) determine the antimicrobial activity of their organic extracts against some MDR pathogenic bacteria and phytopathogenic fungi; (5) determine the total phenolic and flavonoid compounds in the organic extracts of the five isolates showing the highest antimicrobial activity (pathogenic bacteria and fungi), and their antioxidant activity in vitro; (6) evaluate the toxicity of their organic extracts by UV–visible; (7) determine the volatile compound profile of the most active isolate (high antimicrobial and antioxidant activities) by gas chromatography-mass spectrometry (GC–MS) analysis.

## Results

### Physico-chemical analysis of soil samples

The analysis of the soil of site A showed that it is an alkaline (pH = 8.3), not salty (EC = 0.20 ds/m) soil. The texture diagram allowed us to classify it as a sandy-loam soil which is poor in clay (19.97%), has a normal amount of total nitrogen (0.15%), and is moderately rich in organic matter (3.48%). In addition, our findings revealed the presence of exchangeable cations such as K, Mg, Al, Ca, and Si. Mineral elements including O, Fe, and Si were present in the highest concentrations followed by Al, Ca, K, and Mg (Table 1 and Supplementary Fig. S1a). The soil at site B is an alkaline, sandy (90.01%), not salty soil. It is relatively clay- and silt-poor and has a normal content of total nitrogen. It is rich in organic matter and poor in mineral elements except for O, Ca, and Si which were present in high concentrations (Table 1 and Supplementary Fig. S1b). The soil of site C has a slightly alkaline pH, is not salty, and has a slightly elevated total nitrogen contents. It is rich in organic matter (7.61%) and moderately rich in mineral elements (K, Mg, Si, etc.); however, O, Si, and Fe were detected in high concentrations (Table 1 and Supplementary Fig. S1c). The data of the soil pH values in the three sites showed some differences among the different soil textures. The lowest value (pH = 7.29) was recorded in the sandy-silty texture (site A), while the highest (pH = 8.57) was recorded in the sandy texture. The highest values of organic carbon contents were recorded for sites with a sandy-silty texture (sites B and C).

**Table 1:** Physico-chemical analysis of soil samples.

Physico-chemical parameters	Site A	Site B	Site C
Textural soil types	Sandy-silty	Sandy	Sandy-silty
Clay (%)	19.97	5.00	9.99
Sandy (%)	50.08	90.01	55.04
Silt (%)	29.95	4.99	34.97
pH	8.30	8.57	7.29
EC (ds/m)	0.20	0.30	0.15
OM (%)	3.48	5.65	7.61
TN (%)	0.15	0.24	0.33
Mg (%)	1.09	0.31	1.59
Al (%)	5.91	1.08	6.70
K (%)	1,59	2.79	1.39
Ca (%)	2,83	23.00	0.74
S (%)	0.11	0.03	0.01
Cl (%)	0.15	0.04	–
P (%)	0.31	0.00	0.36
Fe (%)	14.80	1.63	12.58
Mn (%)	0.13	0.04	0.20
Cu (%)	0.03	–	0.01
Zn (%)	0.01	0.01	0.03

Site A (Sebt Jahjouh El Hajeb); Site B (Forest of Ain Vittel Ifrane); Site C (Forest of Azrou);

(–) not detectable, *EC* electrical conductivity, *OM* organic matter, *TN* total nitrogen.

### Isolation of *Actinomycetes* strains

A total of 44 phenotypically different presumptive *Actinomycetes* strains were isolated from soil samples collected in the three studied sites, including 11 strains (25%) from site A, 14 strains (31.82%) from site B, and 19 strains (43.18%) from site C (Table 2). The maximum number of isolates was recorded in the M2 medium (n = 20) followed by BEN (n = 11), GLM (n = 8), and GA (n = 4) (Table 2).

**Table 2:** Total number of *Streptomyces* isolates obtained from the three studied sites (A, B and C) according to the culture media used.

Collection site of soil samples	Number of *Actinomycetes* colonies in different isolation media (× 10^3^ CFU/mL)				Total number of *Streptomyces* colonies (× 10^3^CFU/mL)	Number of colonies with distinct morphological characteristics in each medium				Total number of isolates
	M2	Ben	GLM	GA		M2	Ben	GLM	GA	
Site A	80	9	16	12	117	4	2	4	1	11
Site B	60	26	14	4	104	7	4	2	1	14
Site C	45	12	14	14	85	9	4	3	2	18
Total	185	47	44	30	306	20	11	9	4	43

Site A (SebtJ ahjouh El Hajeb); Site B (Forest of Ain Vittel Ifrane); and Site C (Forest ofAzrou).

### Screening of active *Actinomycetes* isolates

**Figure 1:**
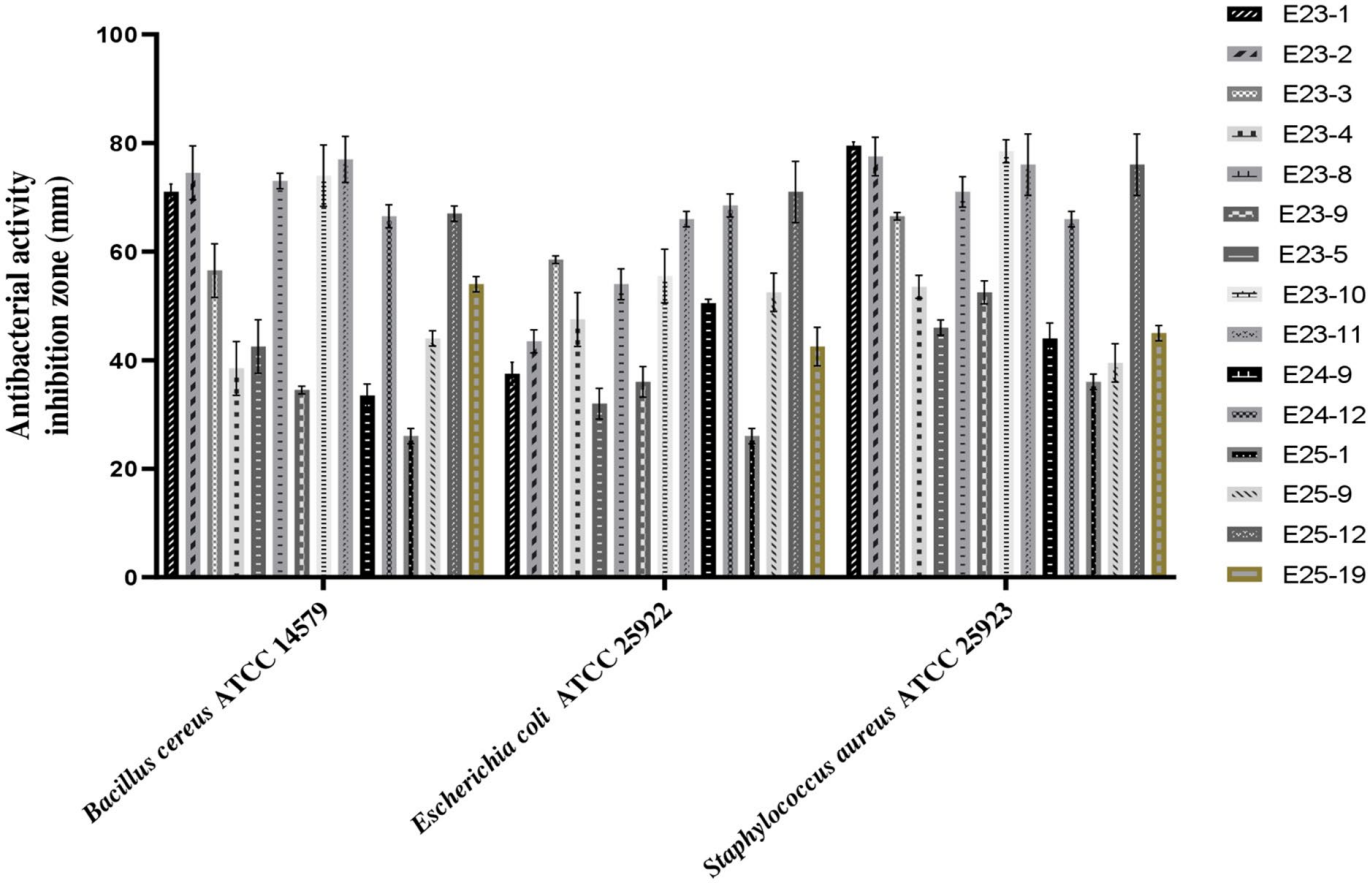
Primary screening (antibacterial activity) using the double layer method on ISP2 medium.

**Figure 2:**
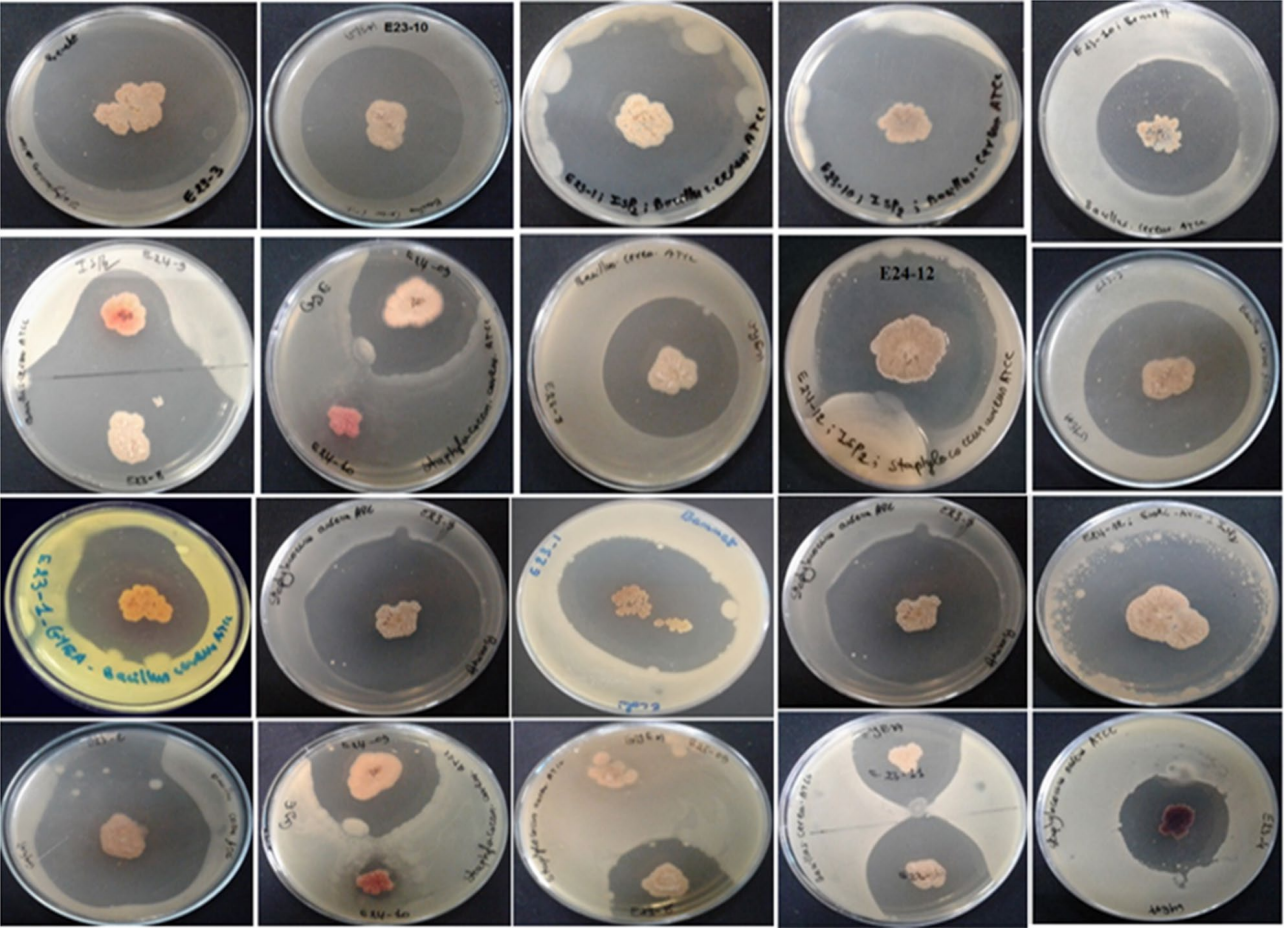
Antibacerial activity of pure *Actinomycetes* isolates by the double layer method on ISP2 medium.

**Figure 3:**
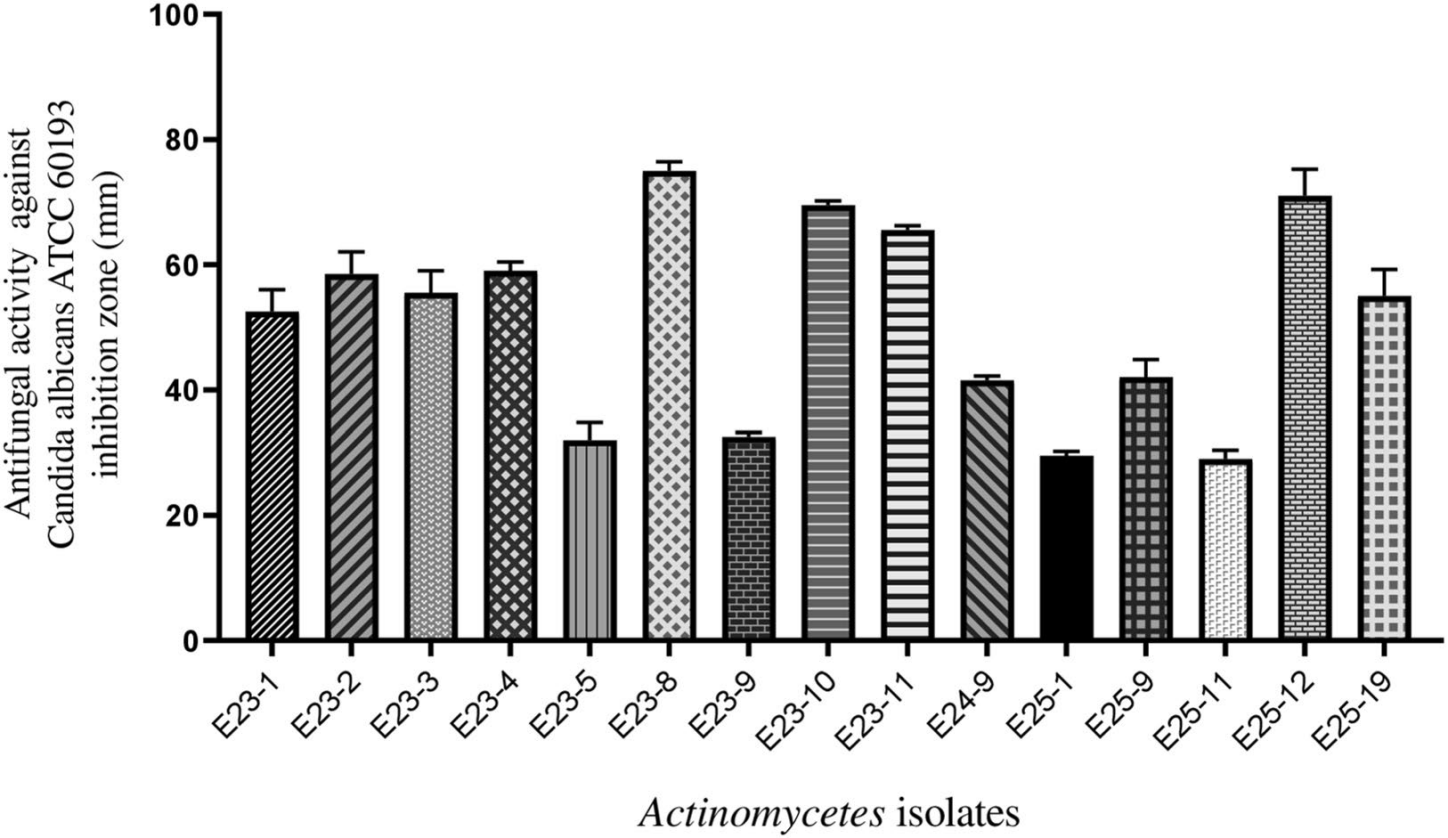
Primary screening (antifungal activity) by the double layer method on ISP2 medium against *Candida albicans* ATCC 60,193.

**Figure 4:**
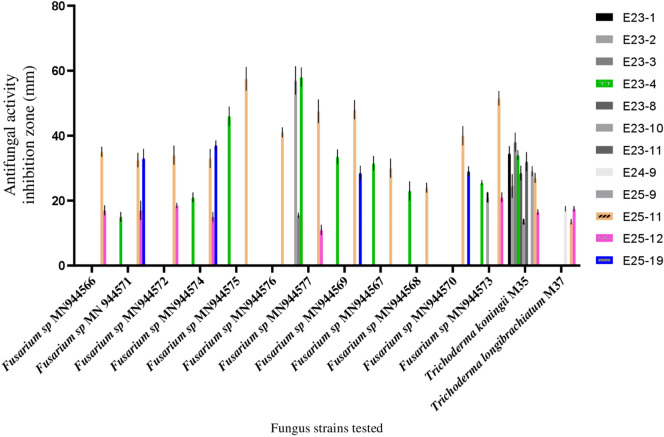
Primary screening (antifungal activity) by the double layer method on ISP2 medium against phytopathogenic fungi.

**Figure 5:**
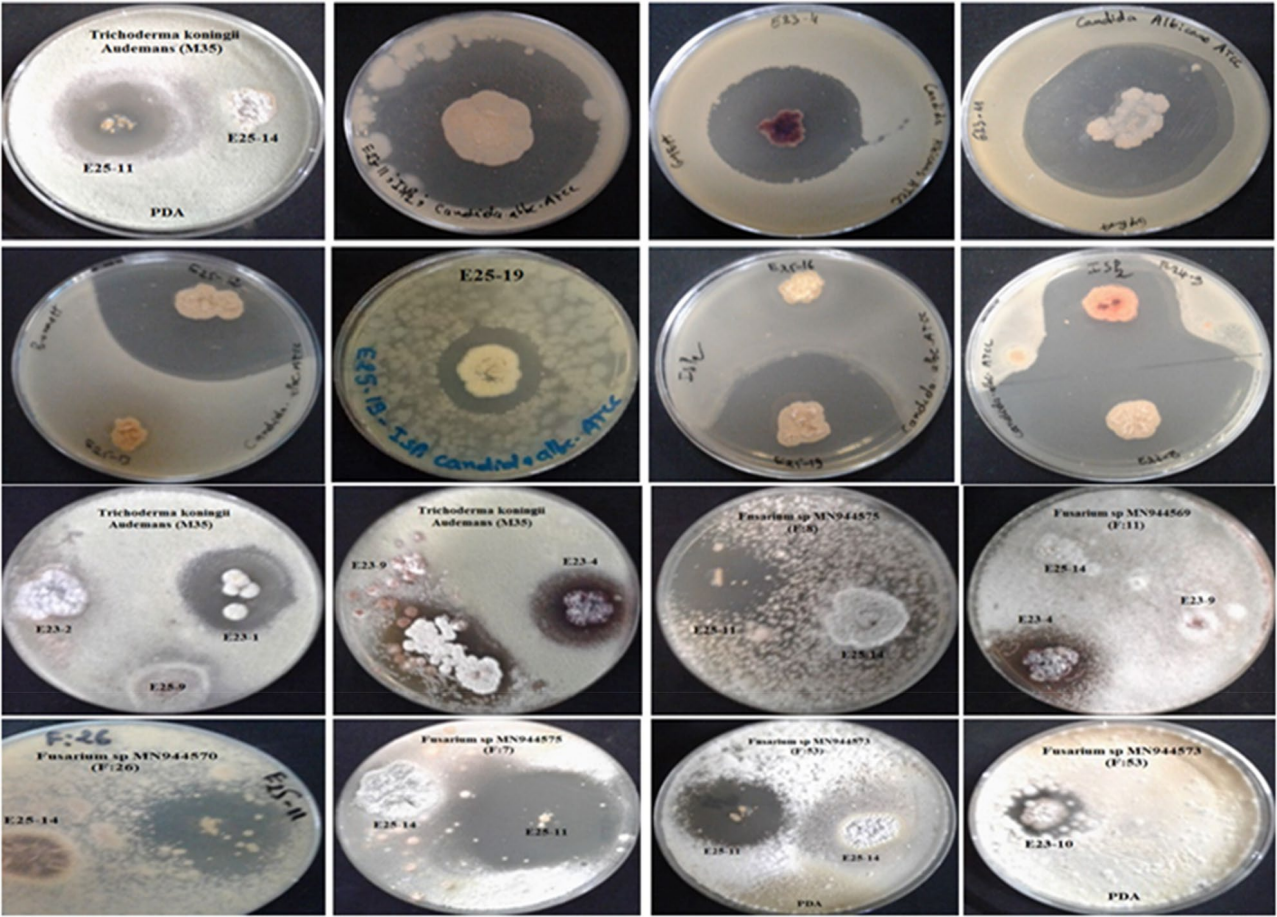
Antifungal activity of *Actinomycetes* pure isolates by the double layer method on ISP2 medium.

**Figure 6:**
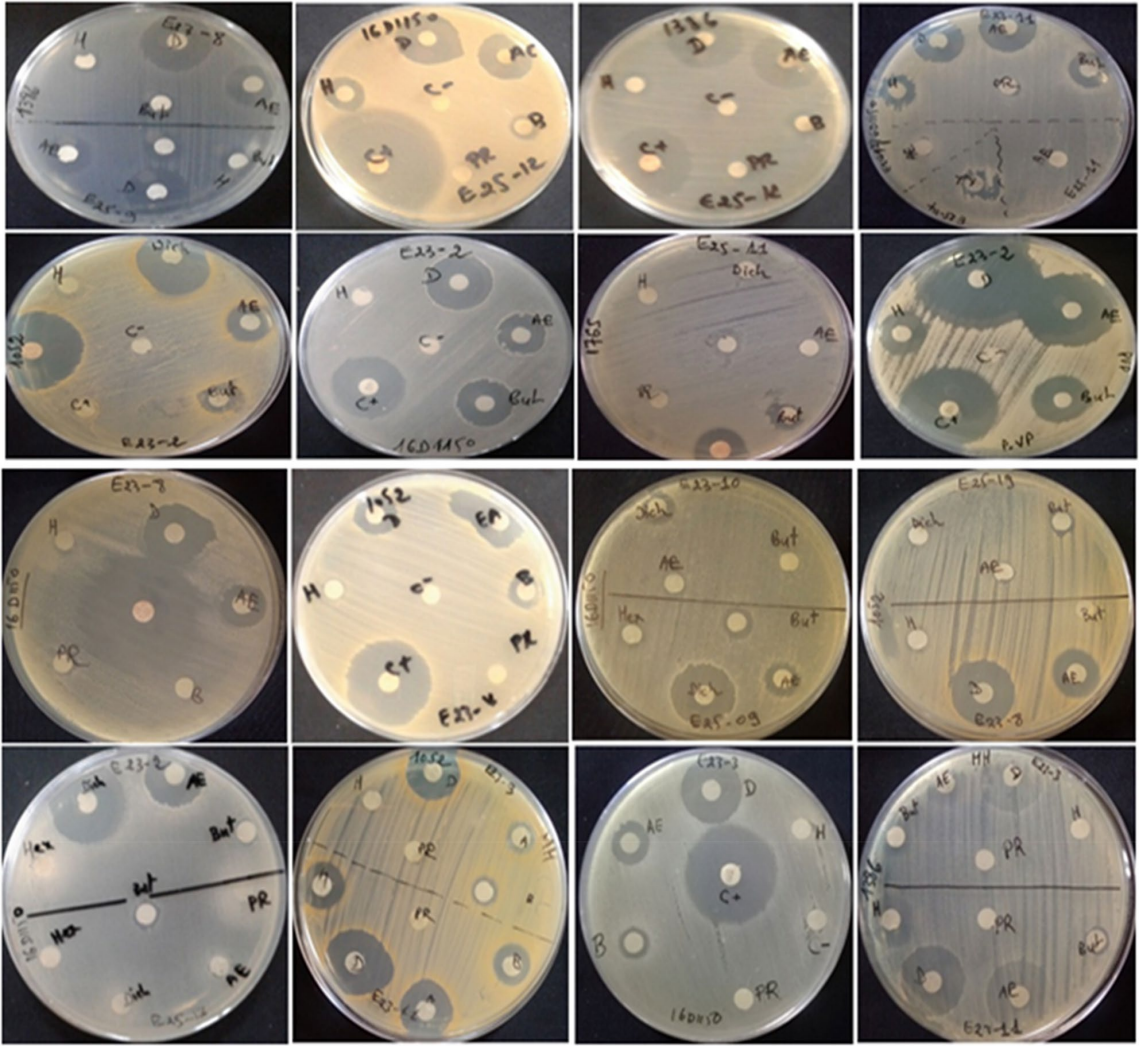
Secondary screening (antibacterial activity) by the disc diffusion method on MH medium.

**Figure 7:**
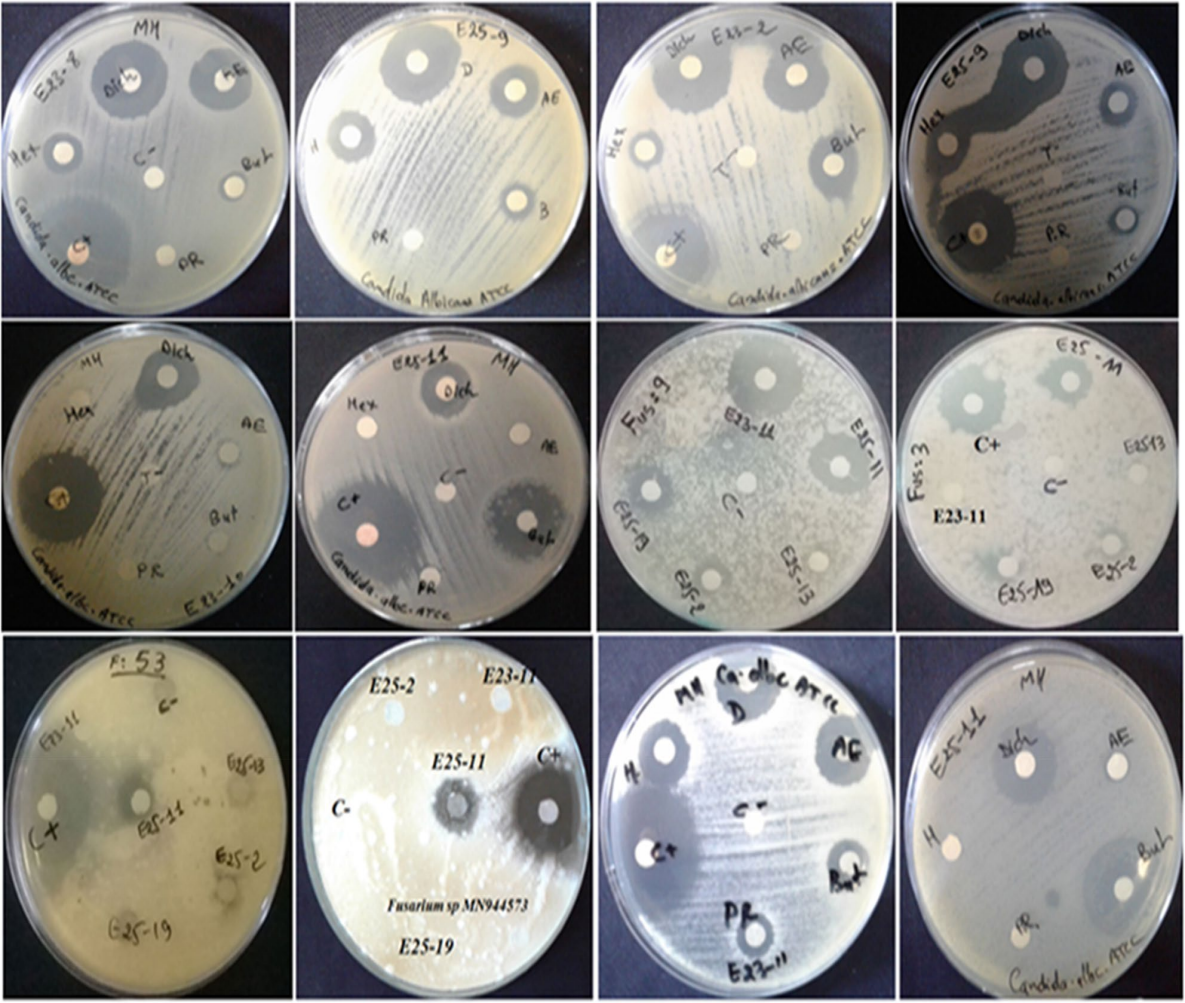
Secondary screening (antifungal activity) by the disc diffusion method on MH medium.

Among the 44 *Actinomycetes* isolates tested, only 16 isolates (37.21%) exhibited moderate to strong activity against Gram-positive bacteria (*Bacillus cereus*ATCC 14,579, *Staphylococcus aureus* ATCC 25,923), Gram-negative bacteria (*Escherichia coli* ATCC 25,922) (Figs. 1 and 2), and all the fungal strains tested (Figs. 3, 4, and 5). These 16 bioactive isolates were subjected to secondary screening against some MDR pathogenic bacteria (Table 3, Fig. 6), as well as phyto-pathogenic fungi (Table 4, Fig. 7).The results of the primary and secondary screening showed that most of the isolates tested were more active against Gram-positive bacteria than Gram-negative bacteria (Figs. 1, 2 and 6, Table 3).

**Table 3:** Secondary screening (antibacterial activity) by disc diffusion method.

Test strains	Dichloromethane extracts of bioactive isolates (inhibition zones in mm)											
	E23-2	E23-3	E23-4	E23-8	E23-9	E23-10	E23-11	E24-9	E25-9	E25-11	E25-12	PC
Clinical *Escherichia coli*16D1150	22.5 ± 0.71	18 ± 2.12	22 ± 2.83	15.5 ± 0.71	21.5 ± 2.12	11.5 ± 0.71	15 ± 1.41	10.5 ± 0.72	19.5 ± 0.70	7 ± 0.0	23.5 ± 2.1	27 ± 1.41
*Clinical Proteus vulgaris* 16C1737	25.5 ± 0.71	–	28 ± 1.41	22 ± 1.41	19 ± 1.41	22.5 ± 0.71	26.5 ± 2.12	17.5 ± 3.54	36.5 ± 2.12	14.5 ± 0.71	35.5 ± 2.12	35.5 ± 0.71
Clinical *Neisseria gonorhae*16D1170	–	–	–	8 ± 0	8 ± 1.41	–	–	–	7.5 ± 0.71	–	7.5 ± 0.71	25.5 ± 2.12
*Escherichia coli* ATCC 25,922	25.5 ± 0.71	–	25 ± 1.41	10 ± 0.0	13.5 ± 2.12	19 ± 1.41	20.5 ± 0.71	12.5 ± 0.71	21.5 ± 0.71	18 ± 1.41	–	25.5 ± 0.71
Clinical *Saphylococcus aureus* 18k1052	22.5 ± 0.71	17.5 ± 0.7	19 ± 1.41	14.5 ± 0.71	8.5 ± 0.71	11 ± 2.83	18 ± 1.41	10.5 ± 0.72	16.5 ± 0.71	–	23 ± 2.83	24 ± 1.41
*Clinical Enterococcus faecalis*18k1386	25 ± 1.41	18.5 ± 2.12	20.5 ± 0.71	14.5 ± 0.71	8.5 ± 0.71	10 ± 1.41	19 ± 1.41	7 ± 0.00	19.5 ± 0.71	–	25 ± 1.41	11 ± 1.41
*Staphylococcus aureus* ATCC 25,923	24.5 ± 3.53	–	21.5 ± 0.71	13.5 ± 0.71	10.5 ± 0.71	16.5 ± 2.12	19.5 ± 0.71	13 ± 1.41	22.5 ± 0.71	17.5 ± 0.71	17.5 ± 0.71	27.5 ± 2.12
*Bacillus cereus* ATCC 14,579	24 ± 1.41	–	23 ± 0.0	14.5 ± 0.71	10.5 ± 0.71	14.5 ± 0.71	21.5 ± 0.10	14 ± 0.0	22 ± 2.82	19.5 ± 4.95	19 ± 1.41	28 ± 4.24

(–) no inhibition zone, *PC* positive control (Streptomycine) and disc diameter = 6 mm.

**Table 4:** Secondary screening (antifungal activity) by disc diffusion method.

Test strains	Dichloromethane extracts of active isolates (inhibition zone in mm)									
	E23-2	E23-3	E23-4	E23-8	E23-10	E23-11	E25-9	E25-11	E25-12	PC
*Candida albicans ATTC* 60,193	22.5 ± 0.71	–	21.5 ± 0.71	125 ± 0,71	17.5 ± 2.12	19.5 ± 0.71	22 ± 1.41	16.5 ± 2.12	20.5 ± 0.71	27 ± 2.83
*Fusarium* sp. MN94457	18.5 ± 2.12	22 ± 2.82	–	13.5 ± 2.12	–	22 ± 1.41	–	19 ± 1.41	–	31.5 ± 0.71
*Fusarium* sp. MN944571	–	–	–	–	–	20.5 ± 0.71	–	14.5 ± 0.71	–	19.5 ± 0.71
*Fusarium* sp. MN944567	11 ± 1.41	–	–	–	–	–	–	–	–	28.5 ± 2.12
*Fusarium* sp. MN944568	–	–	16 ± 2.82	–	–	–	–	–	–	27 ± 1.41
*Fusarium* sp. MN944569	–	10.5 ± 0.71	–	–	–	14.5 ± 0.71	–	9.5 ± 0.71	–	27.5 ± 0.71
*Fusarium* sp. MN944570	–	9.5 ± 0.71	–	–	–	19.5 ± 2.12	–	–	–	26.5 ± 0.71
*Trichoderma koningii* (M35)	–	–	–	–	–	21 ± 1.41	–	16.5 ± 2.12	–	16.5 ± 2.12

(–) no inhibition zone, *PC* positive control (Cycloheximide) and disc diameter = 6 mm.

### Production kinetics of secondary metabolites

**Figure 8:**
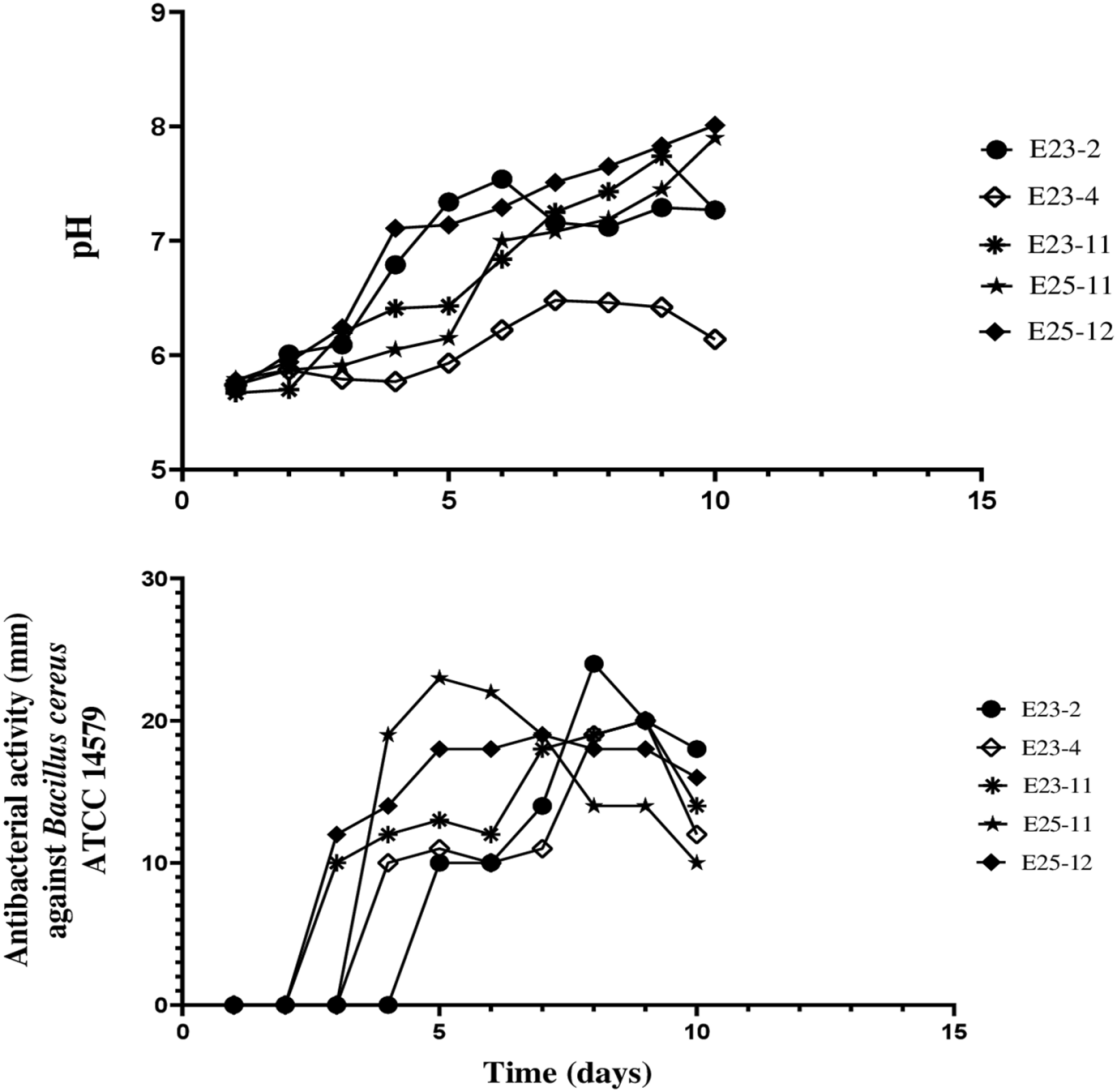
Kinetics of pH evolution and production of antibiotics against *Bacillus cereus* ATCC 14,579.

The antibiotic production kinetics and pH evolution performed by the well diffusion method on the ISP2 liquid medium are shown in Fig. 8 and Supplementary Fig. S2. It was noticed that the antibacterial activities started two days after incubation for the E25-12 and E23-11 strains, three days after incubation for the E25-11 and E23-4 strains, and after four days for the E23-2 strain. During the incubation period, the kinetics of pH evolution showed a slight variation between 5.67 and 8.01.

### Cultural, micro-morphological, biochemical and physiological characteristics of isolates

Among the 16 bioactive isolates, 11 showed strong antimicrobial activity and were selected for taxonomic, physiological, and biochemical studies. The results are presented in Table 5 (as well as in Supplementary Figs. S3 and S4, and Supplementary Table S1). *Actinomycetes* isolates grew very well on all tested media (ISP1, ISP2, ISP4, ISP5 and GYEA). Melanin pigment production, characterized by a dark brown coloration, was observed on the ISP9 base medium complemented by carbonaceous substrates (glucose, melezitose, manitol, trehalose, cellobiose, sucrose, raffinose, xylose, melibiose, mannose, fructose, galactose, maltose, and ribose).

**Table 5:** Cultural, micro-morphological, biochemical and physiological characteristics of *Actinomycetes* isolates.

Characteristics	E23-2	E23-3	E23-4	E23-8	E23-9	E23-10	E23-11	E24-9	E25-9	E25-11	E25-12
**Assimilation**											
Ribose	−	+	+	−	−	−	−	2 +	+	−	+
Melezitose	+	3 +	−	−	−	−	+	−	−	−	−
Manitol	3 +	3 +	3 +	3 +	−	2 +	3 +	−	3 +	3 +	3 +
Trehalose	3 +	3 +	3 +	3 +	3 +	3 +	3 +	3 +	3 +	3 +	3 +
Cellobiose	3 +	3 +	3 +	3 +	3 +	3 +	3 +	3 +	3 +	3 +	3 +
Sucrose	3 +	3 +	3 +	3 +	3 +	2 +	3 +	3 +	3 +	−	3 +
Raffinose	3 +	3 +	3 +	3 +	3 +	2 +	3 +	−	3 +	3 +	3 +
Xylose	3 +	3 +	+	3 +	+	3 +	2 +	3 +	3 +	3 +	3 +
Melibiose	3 +	3 +	3 +	3 +	−	+	3 +	−	3 +	3 +	3 +
Mannose	3 +	3 +	3 +	3 +	3 +	2 +	3 +	3 +	3 +	3 +	3 +
Fructose	3 +	2 +	3 +	3 +	3 +	2 +	3 +	+	3 +	3 +	3 +
Galactose	3 +	3 +	3 +	+	3 +	3 +	3 +	+	3 +	3 +	3 +
Maltose	3 +	2 +	2 +	2 +	2 +	+	3 +	3 +	2 +	3 +	3 +
Glucose	3 +	3 +	3 +	3 +	3 +	3 +	3 +	3 +	3 +	3 +	3 +
**Growth on**											
ISP1	3 +	3 +	2 +	3 +	3 +	2 +	2 +	3 +	2 +	3 +	3 +
ISP2	3 +	3 +	3 +	3 +	3 +	3 +	2 +	3 +	3 +	3 +	3 +
ISP4	3 +	3 +	3 +	3 +	3 +	3 +	2 +	3 +	2 +	3 +	3 +
ISP5	3 +	3 +	3 +	3 +	3 +	3 +	2 +	3 +	3 +	3 +	3 +
ISP7	3 +	3 +	3 +	3 +	3 +	3 +	+	3 +	2 +	3 +	3 +
GYEA	3 +	3 +	3 +	3 +	3 +	3 +	3 +	3 +	2 +	3 +	3 +
**pH tolerance**											
4,63	−	−	−	−	−	−	−	−	−	−	−
5,33	2 +	+	+	+	+	2 +	+	+	+	2 +	+
6,41	2 +	3 +	3 +	3 +	2 +	2 +	2 +	+	2 +	3 +	2 +
7,31	3 +	3 +	2 +	3 +	3 +	3 +	2 +	2 +	3 +	3 +	3 +
8,28	3 +	3 +	3 +	3 +	3 +	3 +	3 +	3 +	3 +	3 +	3 +
9,27	3 +	2 +	2 +	3 +	3 +	3 +	3 +	3 +	3 +	2 +	3 +
10,03	2 +	2 +	+	3 +	+	2 +	3 +	3 +	+	2 +	3 +
**NaCl tolerance**											
1%	3 +	3 +	3 +	3 +	3 +	3 +	3 +	3 +	3 +	3 +	3 +
2%	3 +	3 +	3 +	3 +	2 +	3 +	3 +	3 +	3 +	3 +	3 +
3%	3 +	3 +	2 +	3 +	+	3 +	3 +	2 +	3 +	3 +	3 +
4%	3 +	3 +	2 +	2 +	+	3 +	2 +	+	3 +	3 +	2 +
5%	2 +	3 +	+	+	+	2 +	2 +	+	2 +	3 +	2 +
7%	2 +	+	−	+	−	3 +	+	−	−	−	+
10%	−	−	−	−	−	−	−	−	−	−	−
**Growth on**											
4 °C	−	−	−	−	−	−	−	−	−	−	−
28 °C	3 +	3 +	3 +	3 +	3 +	3 +	3 +	3 +	3 +	3 +	3 +
37 °C	3 +	3 +	+	2 +	2 +	2 +	2 +	3 +	+	3 +	2 +
46 °C	−	−	−	−	−	−	−	−	−	−	−

(−) no growth; (+) low growth; (2+) intermediate growth; (3) good growth.

### Hierarchical cluster analysis

**Figure 9:**
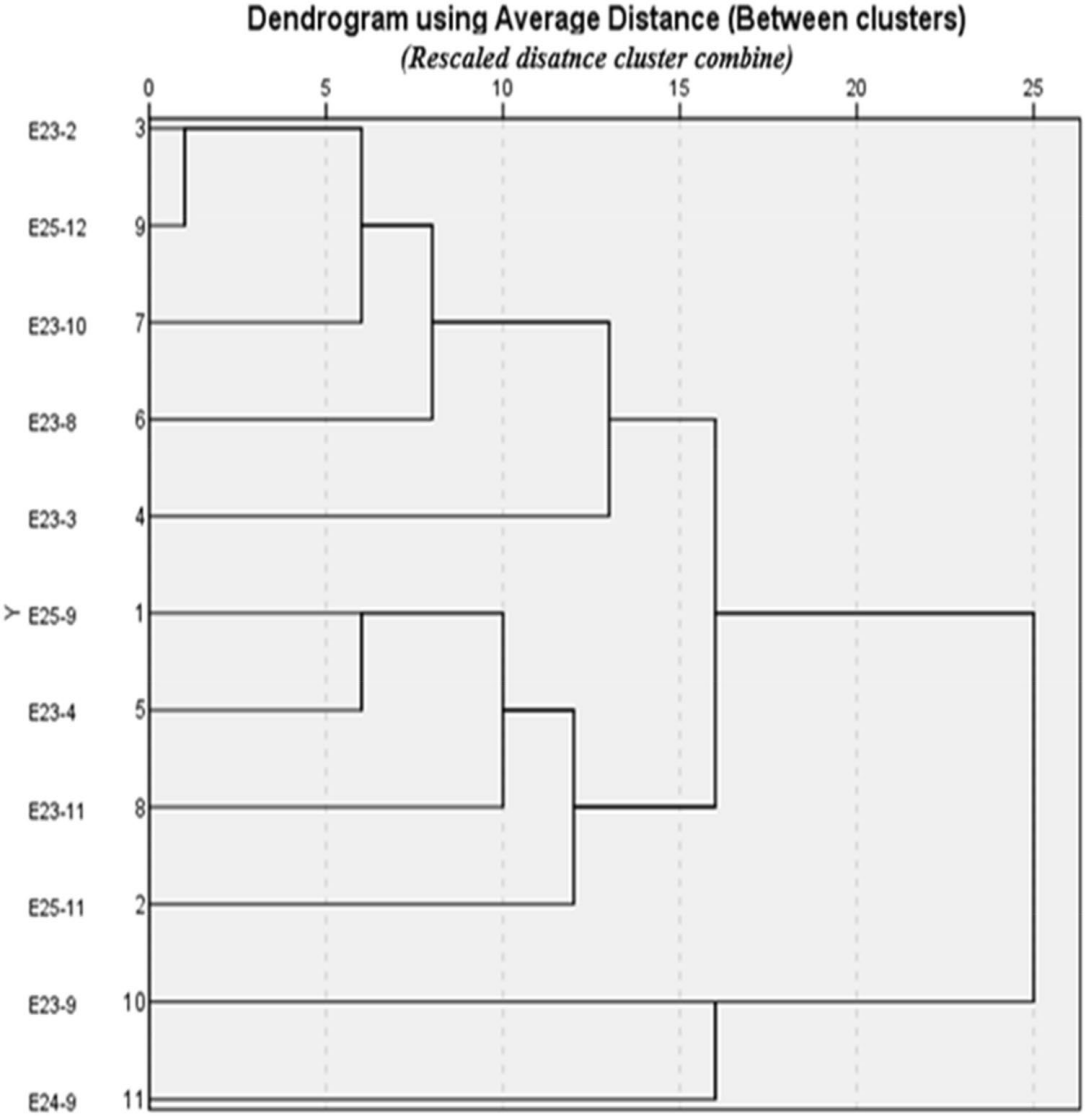
Dendrogram showing the evolutionary relationship of the *Actinomycetes* isolates.

The 11 strains that showed strong antimicrobial activity in the secondary screening were subjected to a hierarchical cluster analysis based on 43 macroscopic, microscopic, biochemical, and physiological characters. A dendrogram based on the average relationship among groups was created using SPSS version 23 software (IBM SPSS statistics 23). The dendrogram divided the active strains of *Actinomycetes* into two main clusters (Fig. 9). Cluster 1 includes nine strains (E23-2, E23-3, E23-4, E23-8, E23-10, E23-11, E25-9, E2511, and E25-12), while the E23-9 and E24-9 strains formed the second cluster.

### Molecular identification and phylogenetic analysis of bioactive strains

**Figure 10:**
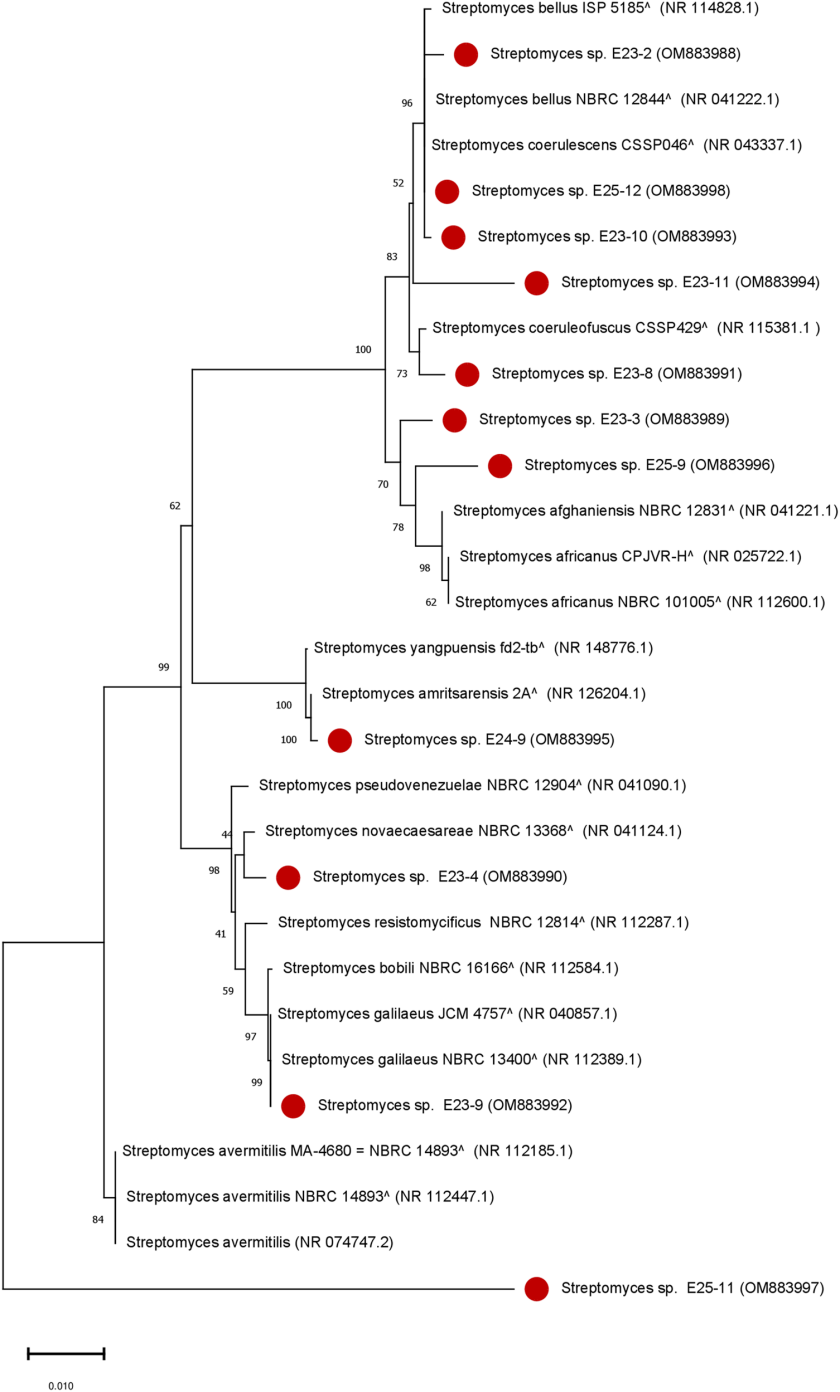
Phylogenetic tree based on 16S rRNA gene sequences of *Actinomycetes* isolates.The phylogenetic tree based on the 16S rRNA gene was established by statistical method (Neighbour-Joining Tree test), showing the evolutionary relationships among the 11 *Actinomycetes* isolates marked in red circle and their closest known taxa. Each branch in the tree is associated with a bootstrap value (a percentage ranging from 0 to 100%) which indicates the number of times it has been found during the repetitions and thus determines its credibility, only the values (> 40%) are displayed. The bar (0.010), represents the number of substitutions per nucleotide position (1% of divergence between sequences). GenBank accession numbers are shown in parentheses.

The 11 active *Actinomycetes* isolates were selected for further characterization using the 16S ribosomal RNA homology sequence study^17^. The Basic Local Alignment Search Tool (BLAST N) program was used to find the closest correspondence in the database of non redundant reference rRNA sequences (refseq_rna). The results indicate that all the isolates tested belong to the genus *Streptomyces* (100%) (Fig. 10). Six isolates were identified up to a species level while the other 5 isolates were identified up to a genus level (Supplementary Table S1). The 5 isolates (E23-3, E23-9, E23-10, E24-9, and E25-12) exhibited a very high percentage of similarity at the species level (> 99%). Isolate E23-3 showed a 99.06% similarity with *Streptomyces africanus* NBRC 101005 T (NR_112600.1), E23-9 was 99.28% similar to *Streptomyces galilaeus* NBRC 13400 T (NR_112389.1), E24-9 showed a 99.63% similarity with *Streptomyces amritsarensis* 2AT (NR_126204.1), while isolates E23-10 and E25-12 were both closely similar to the *Streptomyces bellus* strain NBRC 12844 T (NR_041222.1) (Supplementary Table S2) but differed morphologically.

### Total phenolic and flavonoid contents of crude extracts

The results of the total phenolic and flavonoid contents in the dichloromethane extracts of *Streptomyces* isolates are summarized in Table 6. The results revealed a significant difference (*P* < 0.05) among the tested isolates. The E23-11, E25-11, and E23-4 isolates exhibited the highest phenolic contents among the isolates. Concerning flavonoids, the isolate E23-4 contained the highest flavonoid contents.

**Table 6:** Total phenolic and flavonoid contents of dicholoromethane extract of *Actinomycetes.*

Dichloromethane extracts	Total phenol’s contents (mg GAE/mg extract)	Total flavanoids contents (mg QE/mg extract)
E23-2	0.36 ± 0.012^C^	0.028 ± 0.005^C^
E23-4	0.42 ± 0.004^B^	0.044 ± 0.001^A^
E23-11	0.64 ± 0.008^A^	0.031 ± 0.002^B^
E25-11	0.49 ± 0.016^B^	0.035 ± 0.002^B^
E25-12	0.10 ± 0.001^D^	0.032 ± 0.005^B^

Values expressed are means ± SD (n = 3).

In each column, averaged means within followed by the same letters are not significantly different according to one-way (ANOVA) using Tukey's multiple comparisons test, *P* < 0.05.

### In vitro antioxidant activities

All dichloromethane extracts of *Streptomyces* tested revealed a lower DPPH free radical scavenging activity than ascorbic acid (*P* < 0.01) (Table 7), while no significant difference was recorded among the extracts (*P* > 0.01). Regarding ABTS free radical scavenging activity, the dichloromethane extracts of the tested *Streptomyces* strains exhibited ABTS cationic radical scavenging activity in the range of 29–35% inhibition (*P* < 0.0001) (Table 7). The E23-4 strain extract exhibited the highest activity (35.79%), followed by the E23-2 (33.8%) and E25-11 (32.67%) strains. However, no significant difference was recorded between the E23-11 (29.45%) and E25-12 (29.31%) strains. The highest antioxidant activity (76.38%) was recorded in trolox (positive control) (Table 7).

**Table 7:** Antioxidant activity of isolates dichloromethane extracts in different antioxidant assays (ABTSand DPPH).

*Actinomycetes* isolates	Antioxidant activity	
	ABTS radical Scavenging activity (%)	DPPH radical Scavenging activity (%)
E23-2	33.81 ± 3.12^B^****	21.17 ± 3.18^B^**
E23-4	35.79 ± 0.22^B^****	25.55 ± 0.27^B^ ***
E23-11	29.45 ± 2.06^C^****	21.88 ± 4.19^B^***
E25-11	32.67 ± 0.36^B^****	24.45 ± 2.37^B^**
E25-12	29.31 ± 0.71^C^****	19.95 ± 1.09^B^***
Trolox	66.67 ± 0.74^A^	ND
Ascorbic Acid	ND	76.38 ± 1.82^A^

Values expressed are means ± SD (n = 3).

In each column, averaged means within followed by the same letters are not significantly different according to one-way (ANOVA) using Tukey's multiple comparisons test, *P* < 0.05.

*ND* not determined.

**P < 0.01, ***P < 0.001, ****P < 0.0001 indicates statistically significant difference.

### Correlation of total phenolic and flavonoid contents with antioxidant activities

**Figure 11:**
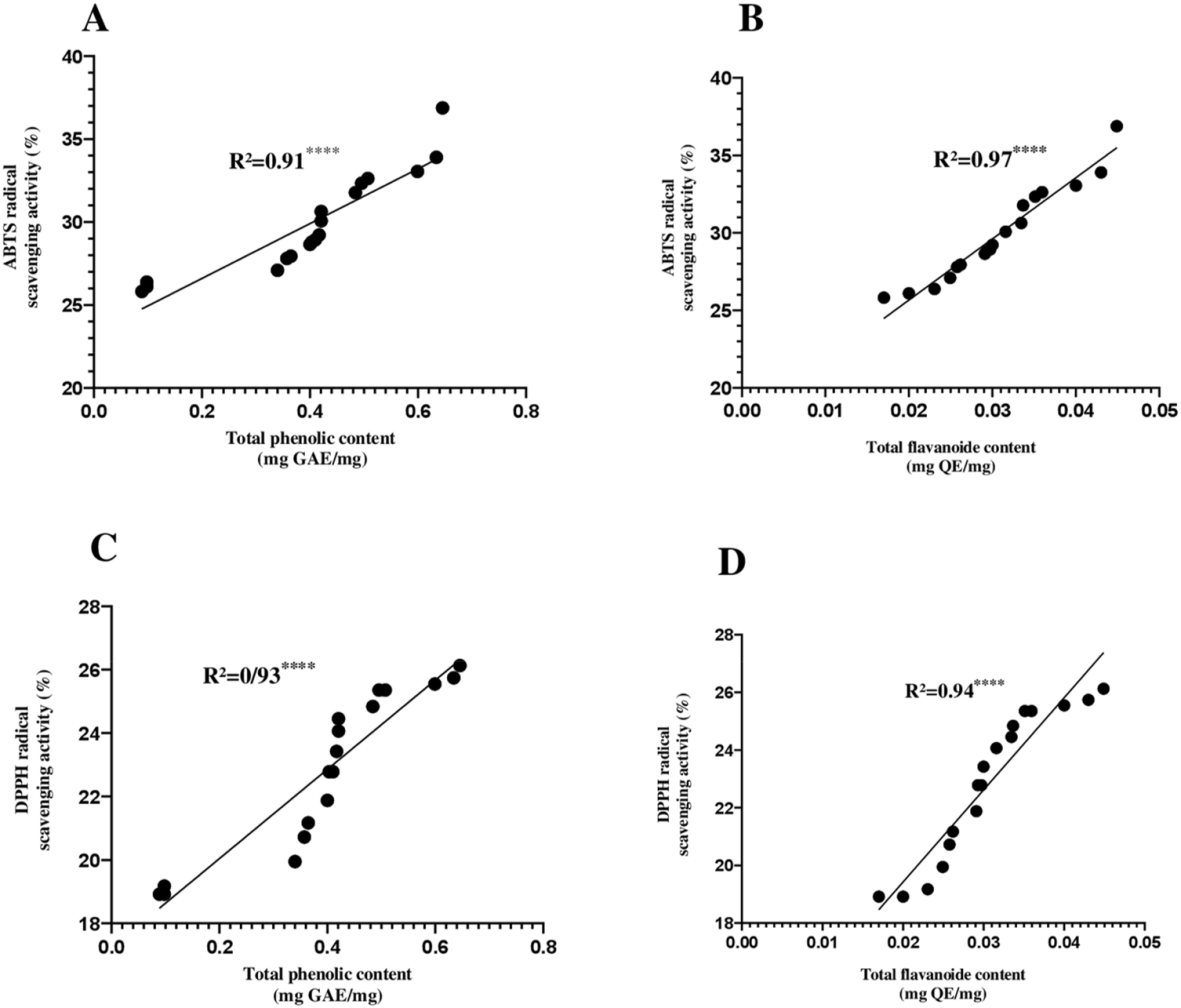
Pearson correlation graphics. (**A**) Pearson correlation between ABTS and total phenolic content, (**B**) Pearson correlation between ABTS and flavonoids content, (**C**) Pearson correlation between DPPH and total phenolic content, and (**D**) Pearson correlation between DPPH and flavonoids content. “****” (*P* < 0.0001) highly significant between tests.

The results revealed a highly significant positive correlation (*P* < 0.0001) between the total phenolic and flavonoid contents and the antioxidant capacity, analyzed by two different assays (ABTS and DPPH). The highest correlation was observed in the ABTS radical scavenging activity (R^2^ = 0.94) (Fig. 11A,B), followed by DPPH radical scavenging activity (R^2^ = 0.93) (Fig. 11C,D).

### UV–visible spectrum analysis of extracts

**Figure 12:**
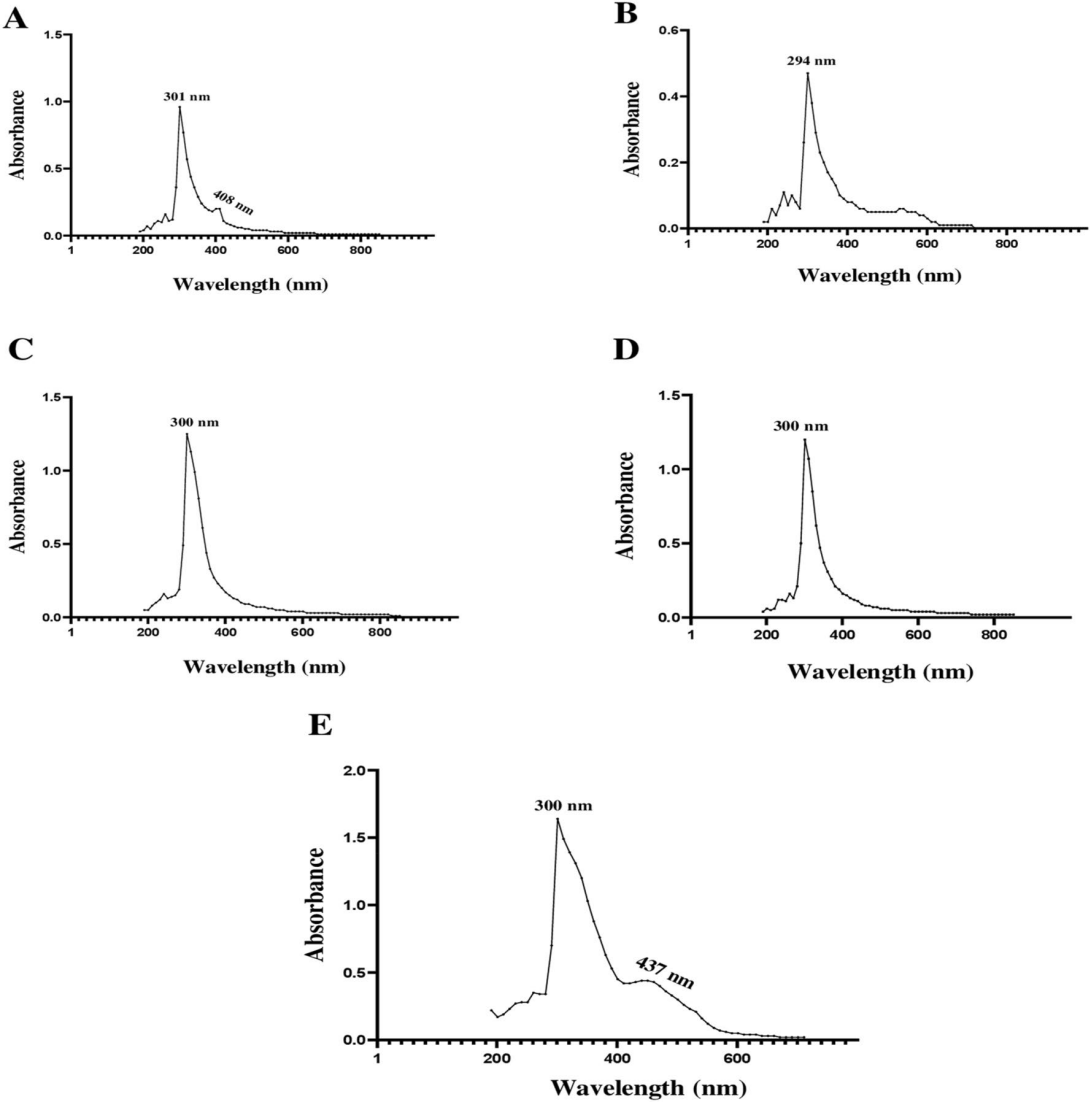
UV–visible spectra for the five active crude dichloromethane extracts. (**A**) Brut extract of *Streptomyces* sp. E23-2 OM883988, (**B**) brut extract of *Streptomyces* sp. E23-4 OM883990, (**C**) brut extract of *Streptomyces* sp. E23-11 OM883994, (**D**) brut extract of *Streptomyces* sp. E25-11 OM883997, (**E**) brut extract of *Streptomyces bellus* E25-12 OM883998.

The absorption spectra of dichloromethane extracts obtained from the five most active *Streptomyces* strains tested did not show the three characteristic absorption peaks of polyenes. Two absorption peaks were obtained at 301 and 408 nm for the E23-2 strain. Only one peak was obtained at 294 nm for the E23-4 strain. Moreover, one peak was obtained at 300 nm for the E23-11 and E25-11 strains, and two peaks were obtained at 300 and 437 nm for the E25-12 strain (Fig. 12).

### Identification of bioactive volatile compounds by GC–MS

The GC–MS analysis of the dichloromethane extract of the E23-4 OM883990 strain which showed a very strong antimicrobial and antioxidant activity compared to the other strains tested allowed us to identify a total of 21 volatile compounds whose elution lasted between 9.197 and 49.381 min. Among these identified compounds, twelve were recognized for their antimicrobial, antifungal, antioxidant, antitumor, and other biological activities (Table 8, Supplementary Figs. S5 and S6). Diethyl trisulfide, Indole-3-carboxylic acid,5-methoxy-2-methyl-1-(3-methylphenyl)-ethyl ester,disulfide, dimethyl, l-Leucine, N-cyclopropylcarbonyl-, pentadecylester; 1,2-Benzisothiazol-3-amine,TBDMS derivative, Pyrrolo[1,2-a] pyrazine-1,4-dione, hexahydro-3-(phenylmethyl), and benzeneacetic acid were the main compounds identified from the dichloromethane extract of the E23-4 OM883990 strain.

**Table 8:** 21 volatile compounds identified by GC–MS from dichloromethane crude extract of *Streptomyces* sp. E23-4 OM883990 strain. RT: Retention time; M.W.: Molecular Weight.

RT (time)	Area (%)	M.W. (g/mol)	Molecular formula	Coompound name	Reported bioactivity
9.197	3.37	94.20	C_2_H_6_S_2_	Disulfide, dimethyl	Antioxidant, Antifungal, Analgesic effect^94–96^
9.366	0.28	85.13	C_3_H_3_NS	1,3-thiazole	Antibacterial activity^97^, anti-inflammatory activity^98^ , antitumor and cytotoxic activity^99^
9.467	1.25	94.10	C_6_H_5_OH	Phenol	Antimicrobial activity, antibacterial activity, and antioxidant activity^100^
9.580	0.30	112.08	C_4_H_4_N_2_O_2_	Maleic hydrazide	Anti-tumorigenic effect^101^
10.267	0.18	126.11	C_6_H_6_O_3_	Methyl 2-furoate	Antifungal^102^
10.583	0.67	126.11	C_6_H_6_O_3_	Maltol	Antioxidant, Anti-inflammatory, and Antitumor^103^
10.955	0.76	144.12	C_6_H_8_O_4_	4H-Pyran-4-one, 2,3-dihydro-3,5-dihydroxy-6-methyl-	Antioxidant^104^
12.746	0.80	136.14	C_8_H_8_O_2_	Benzeneacetic acid	Antifungal, Antimicrobial, and Antioxidant^70–72^
19.767	0.40	222.24	C_12_H_14_O_4_	Diethyl Phthalate	Antimicrobial, Antifungal^105^
24.962	1.57	283.36	C_15_H_25_NO_4_	l-Proline, N-allyloxycarbonyl-, isohexyl ester	Not yet reported
26.866	3.22	409.6	C_25_H_47_NO_3_	l-Leucine, N-cyclopropylcarbonyl-, pentadecyl ester	Antibacterial^106^
27.328	6.21	154.3	C_4_H_10_S_3_	Diethyl trisulphide	Antimicrobial^62^
27.553	1.83	451.68	C_27_H_49_NO_4_	l-Proline, N-allyloxycarbonyl-, octadecyl ester	Not yet reported
34.968	2.77	244.29	C_14_H_16_N_2_O_2_	Pyrrolo [1, 2-a] pyrazine-1, 4-dione, hexahydro-3-(phenylmethyl)	Antifungal, Antioxidant, and Anibacterial^65–67^
47.307	3.54	323.41	C_18_H_17_N_3_OS	Acetamide, N-[4-[2-[(3-methylphenyl) amino]-4-thiazolyl]phenyl]-	Anticancer activity^107^
47.454	4.76	323.39	C_20_H_21_NO_3_	Indole-3-carboxylic acid, 5-methoxy-2-methyl-1-(3-methylphenyl)-, ethyl ester	Anticonvulsant, Antioxidant, Anticancer, Antibacterial and Anti-inflammatory Antitubercular, Antimalarial Resistance mediator against necrotrophic pathogens ^63,64^
47.961	0.22	222.40	C_13_H_22_OSi	Thymol, TMS derivative	Anibacterial, Antifungal, Antioxidant, Anticancer^24,108,109^
48.524	0.36	264.46	C_13_H_20_N_2_SSi	1, 2-Benzisothiazol-3-amine, TBDMS derivative	Antifungal, Antioxidant and Anti-proliferative^68,69^
48.829	0.36	222.40	C_13_H_22_OSi	Thymol, TMS derivative	Anibacterial, Antifungal, Antioxidant, Anticancer^24,108,109^
48.975	0.21	222.47	C_12_H_22_Si_2_	1,2-Bis (trimethylsilyl) benzene	Antimicrobial, Antioxidant, Anibacterial^110^
49.381	0.44	207.27	C_15_H_13_N	Benzo[h]quinoline, 2,4-dimethyl-	anti-inflammatory^111^, antioxidant^112^, anti-HIV^113^, antifungal^114^, treatment of neurodegenerative diseases^115^, antitubercular^116^, and anticancer^117^

## Discussion

### Physico-chemical analysis of soil samples

Several studies have reported that the analysis of soil physico-chemical properties such as texture, pH, salinity, mineral elements, organic matter contents, and total nitrogen could influence soil microbial communities, especially *Streptomyces* spp.*,* which are the most abundant microorganisms identified in soil^18,19^. To understand the impact of ecological conditions on the distribution of *Streptomyces* in different soils, a statistical analysis was performed to determine the correlation between each physico-chemical parameter of the soil and the total number of *Streptomyces* isolated*.* A correlation between these parameters and the total number of *Streptomyces* (TNS) was recorded. There was a significant positive correlation between the TNS and organic matter (r = 1.00**, *P* < 0.01), as well as between TNS and total nitrogen (r = 1.00**, *P* < 0.01). In addition, there was a significant negative correlation between TNS and sulfur (S) (r = − 1.00**, *P* < 0.01), and between TNS and Cl (r = − 1.00**, *P* < 0.01) (Supplementary Table S3). Similar results were previously reported by Dhanasekaran et al.^20^. Moreover, our results were coherent with those of Ghanem et al.^21^, which showed a significant positive correlation between total nitrogen, organic matter, and the TNS isolated*,* while the correlation between pH, temperature, and dissolved phosphates and the TNS isolated revealed non-significant negative values. It can be concluded that although *Actinomycetes* are ubiquitous, their population dynamics are often influenced by the available nutrients and the physico-chemical conditions of the ecosystem.

The total number of isolated *Streptomyces* was higher in sites C and B than site A, reflecting the richness of these two sites (B and C) in organic matter. Lee and Hwang^22^ reported that organic matter contents in soil can also be an important environmental indicator influencing the colonization of *Actinomycetes.* Another study reported that the number of *Actinomycetes* increases with organic matter contents in soil^23^.These results were confirmed by this study, in which a significant positive correlation was found between TNS and organic matter (Supplementary Table S3). Furthermore, it has been reported that soil pH provides selective pressure for bacterial growth^24,25^. Unlike fungi which prefer acidic and humid circumstances, the majority of *Actinomycetes* have been shown to grow best under slightly alkaline conditions^26^. Moreover, Lee and Hwang^22^ reported that the majority of soil *Actinomycetes* are neutrophilic. They grow in a pH range between 7 and 11, with optimal growth at neutral or slightly alkaline pH values (between 7 and 8)^27^. Indeed, they are particularly sensitive to acidity^28^. Consequently, the average pH of our samples was 8.05, which means that pH of the tested soils was slightly alkaline and did not prevent the growth of *Actinomycetes.*

### Pretreatment of soil samples and isolation of *Actinomycetes* strains

The soil samples were pretreated with calcium bicarbonate (CaCO_3_) to reduce the fungal flora and increase the number of probable *Actinomycetes*^29^. According to a previous study^27^, drying soil samples for 7–21 days at ambient temperature can reduce the soil microbial flora because fast-growing bacterial colonies inhibit the growth of other bacteria, including *Actinomycetes*. Hence, in order to isolate *Actinomycetes*, the number of these bacteria (fast-growing bacteria) must be reduced. Moreover, the addition of an antimicrobial agent to culture media has been shown to be effective in removing contaminating microorganisms and thus facilitating the growth of slow-growing *Actinomycetes*^30^.

Isolation was performed on four culture media chosen according to previous studies^30–33^. Compared to the BEN, GLM, and GA media, the M2 medium appeared to be the best for isolating *Actinomycetes*. This result can be explained by the presence of macromolecules (starch and casein) in this medium, which are catabolized by the majority of *Actinomycetes* and make the medium (M2) favorable to the growth of these bacteria^34^. In addition, this medium contains trace elements that are essential for bacterial growth^34^. The GA medium was the least effective in isolating *Actinomycetes*, suggesting that the main component of this medium (L-asparagine) may not provide an adequate nitrogen source for *Actinomycetes* isolated in the Fez-Meknes region.

### Screening of active *Actinomycetes* isolates

The results of the primary and secondary screening revealed that the majority of the tested *Actinomycetes* isolates were more active against Gram-positive bacteria than Gram-negative bacteria. This difference could be explained by the higher complexity of the cell walls of Gram-negative bacteria compared to those of Gram-positive bacteria. The cell walls of Gram-positive bacteria contain lipopolysaccharides (LPS) as their main structural component, which are anchored in the phospholipid layer, making the wall impermeable to lipophilic solutes^35,36^. In contrast, the walls of Gram-positive bacteria mainly consist of peptidoglycan, which does not provide an effective permeability barrier, making Gram-positive bacteria susceptible to metabolites^29,37^. All the tested clinical bacteria were multi-resistant to antibiotics (Supplementary Table S4), but none of these were resistant to the organic extracts of the tested *Actinomycetes* isolates. This suggests that these *Actinomycetes* isolates secrete different antibiotics than those to which the bacteria are resistant.

### Morphological identification of bioactive strains

The production of melanoid pigments is a very important characteristic for *Streptomyces* classification^38^*.* According to Dastager et al.^39^, some *Actinomycetes* can produce dark brown substances in culture media commonly called melanoid pigments. Vinarov et al.^40^ considered that the compounds of this melanoid pigment are irregular polymers which are dark brown in color and formed by many microorganisms via fermentative oxidation. Melanoid pigmentshave a broad spectrum of biological activities serving radioprotective^41^, antioxidant^42^, antimicrobial^43^, and antitumor^44^ functions. *Actinomycetes* strains grow at a pH ranging from 5.33 to 10.03 with an optimum growth at pH 8.28, but they can not grow in acidic media (pH < 5). Hence, it was seen that the *Actinomycetes* isolates tested here tended to grow better under neutral or slightly alkaline pH conditions. Most *Actinomycetes* species are mesophilic with optimal growth temperatures between 25 and 30 °C^8^. The most favorable temperature range for the growth of the tested *Actinomycetes* strains was between 28 and 37 °C. None of the *Actinomycete*s strains tested could grow at 4 °C and 46 °C. These results agree well with those obtained by Singh et al.^45^. All *Actinomycetes* strains tolerate NaCl concentrations from 1 to 5%, without any growth beyond 7% (Table 5). These results suggest that the selected *Actinomycetes* strains had the ability to resist NaCl concentrations up to 70 g/L (7%). Tian et al.^43^ concluded that saline or hyper-saline environments require special consideration because they could provide new avenues for the discovery of new natural and industrially useful molecules.

### Molecular identification and phylogenetic analysis of bioactive strains

The results of the molecular identification based on the 16S ribosomal RNA homology are in agreement with previous studies and confirm that *Streptomyces* are the predominant genus in soils compared to the other *Actinomycetes* genera^46,47^. In fact, Kitouni et al.^47^ reported that 93% of *Actinomycetes* active strains belong to the *Streptomyces* genus.

In this study, three strains (E23-11, E25-9, and E25-11) were found to have a similarity of at least 98.7% with the closest known strains, which implies that they could be new taxa^48^. These results were confirmed with other databases (EzBioCloud) and yielded the same similarities with the same closest taxa (90.24% for E25-11, 97.25% for E23-11, and 98.20% for E25-9). The phylogenetic tree analysis of the selected strains showed that the proximity or distance among these strains were independent of the profile of macroscopic, microscopic, biochemical, and physiological characters. For example, phylogenetically, the E23-4 and E24-9 strains were quite similar, but considerable differences were found between their macroscopic, physiological, and biochemical characteristics, as well as antimicrobial activity. The dendrogram revealed that these two strains were distinct. It was recalled that the E23-4 strain was isolated from the Sebt Jahjouh El Hajeb site, and the second strain (E24-9) was isolated from the Ain Vittel Ifrane forest site, and therefore, the difference in natural habitats (origin of isolation of the strains) could be the cause of this independence. These results agree with those reported by Sengupta et al.^49^.

### In vitro antioxidant activities

The uncontrolled production of oxygen-free radicals causes oxidative stress, which has been identified as a major cause of health problems such as cancer and other diseases^50^. Antioxidants can reduce the accumulation of free radicals in the body, protecting it from oxidative damage^51^. The *Streptomyces* bacteria genus has been recognized as one of the major sources of natural antioxidant compounds^52–55^. However, evidence suggests that the use of a single test may not be sufficient to determine the antioxidant activity of extracts^56^. Therefore, two in vitro assays were performed (ABTS and DPPH). The results of DPPH and ABTS free radical scavenging activities revealed a strong antioxidant capacity of the *Streptomyces* strains tested, which may suggest that these strains may be able to produce one or more antioxidants that could be useful to prevent oxidative stress. At a concentration of 1 mg/mL, the dichloromethane extract of the E23-4 OM883990 strain showed the highest free radical scavenging activity for both ABTS and DPPH assays. Using the same concentration, Tan et al.^57^ obtained DPPH (12.03%) and ABTS (27%) for a *Streptomyces* sp. MUM212 strain dichloromethane extract from Malaysia. Previous studies have shown that the *Streptomyces* genus can produce natural products such as phenols, flavonoids, steroids, and other compounds that are known for their antioxidant activity^55,56,58^. Phenolic compounds are known for their antioxidant activity as well as other bioactivities such as anti-inflammatory, antimicrobial, and anti-allergic properties^59,60^.The statistical analysis in this study supports this hypothesis since a strong correlation was found between antioxidant activity and total phenolic and flavonoid contents.

### Detection of polyene molecules by UV–vis

The analysis of the spectra of the active dichloromethane extracts of the five *Streptomyces* sp. strains showed high antimicrobial activity indicating that they do not contain molecules of a polyene nature, which are characterized by three characteristic absorption maxima in the UV–visible between 291 and 405 nm^61^. This result is very interesting, as polyene molecules are usually discarded in research programs on new bioactive molecules due to their toxicity. It was demonstrated that antifungal molecules of a polyene nature interact with cholesterol since the latter has a structure close to that of ergosterol, the main sterol of fungal cells. This may explain the toxicity of this type of antifungal molecules^57^.

### Identification of bioactive volatile compounds by GC–MS

In this study, the GC–MS analysis of the dichloromethane extract of the E23-4 OM883990 strain revealed the presence of 21volatile compounds with seven majors ones including: diethyl trisulfide, which was found to have a high retention time as well as antibacterial and antifungal activities^62^; indole derivatives (Indole-3-carboxylic acid, 5-methoxy-2-methyl-1-(3-methylphenyl)-, ethyl ester), which exhibit various biological activities namely anticonvulsant, antioxidant, anticancer, antibacterial, anti-inflammatory, antitubercular, and antimalarial activities^63,64^; pyrrolizidine derivatives (Pyrrolo [1,2-a]pyrazine-1,4-dione, hexahydro-3-(phenylmethyl)), which are natural heterocyclic compounds known to exhibit antifungal, antioxidant, and antibacterial activities^65–67^; 1,2-Benzisothiazol-3-amine, a TBDMS derivative which is one of the tert-butyldimethylsilyl (TBDMS) derivatives that are considered an antifungal, antioxidant, and antiproliferative bio-molecule^68,69^; and benzeneacetic acid, which showed antifungal activity against phytopathogens^70–72^. It is possible that all compounds recorded in the tested E23-4 strain dichloromethane extract were responsible for the diversity of biological activities exhibited by this extract. In addition, the highest antimicrobial and antioxidant activities exhibited by the E23-4 strain may have been caused by the synergistic effect of the secondary metabolites present in the E23-4 dichloromethane extract. Our results are in agreement with those found by Das et al.^47^, who showed that the ethyl acetate extract of the *Streptomyces* sp. strain EA-PWS52 consists of bioactive molecules such as benzene acetic acid, pyrrolizidine derivatives, heterocyclic compounds, and other compounds that can exhibit antibacterial, antifungal, and antioxidant activities^30^.

## Conclusions

In conclusion, the *Streptomyces* genus remains an excellent source for the production of natural bioactive compounds, since two thirds of all the new bioactive compounds discovered from *Actinobacteria* between 2015 and 2019 were obtained from the *Streptomyces* genus^14^. This study describes the isolation of *Actinomycetes* strains, particularly the *Streptomyces* genus, from an extremely cold and microbiologically unexplored terrestrial environment of northwestern Morocco. The *Streptomyces* sp. E23-2 OM883988, E23-4 OM883990, E23-11OM883994, E25-11OM883997 and *Streptomyce sbellus* E25-12 OM883998 strains showed remarkable antimicrobial activity against MDR strains and phytopathogenic fungi. These strains can also scavenge several free radicals, including DPPH and ABTS radicals. The phenolic and flavonoid compounds present in their extracts could be the main constituents responsible for their antioxidant properties. The different bioactive compounds identified using GC–MS in the E23-4 dichloromethane extract of *Streptomyces* sp.OM883990 have a wide spectrum of pharmacological activities and can be used to treat bacterial infections multi-resistant, and to counteract diseases caused by oxidative stress such as cancer, cardiovascular diseases, renal diseases, and neurological diseases. These identified bioactive compounds require further studies regarding their mechanisms of action at the intracellular level. In addition, future directions would be to identify and isolate the most interesting compounds in terms of antimicrobial, insecticidal, antioxidant, and antitumor screening using Fourier Transform Infrared Spectroscopy (FTIR), High Performance Liquid Chromatography (HPLC), High Resolution Mass Spectrometry (HRMS), and reconfirming the structure using Nuclear Magnetic Resonance (NMR). Also, further studies regarding the pathways involved in the antimicrobial and antioxidant properties of the identified compounds are needed.

## Materials and methods

### Collection of soil samples

The soil samples were collected during February and early March, 2019 in the Northwest of three different habitats in Morocco including Sebt Jahjouh El Hajeb (GPS: 33° 41′ 16″ N, 5° 22′ 15″ W), forest Ain Vittel Ifrane (GPS: 31° 42′ 07″ N, 6° 20′ 57″ W), and forest of Azrou (GPS: 33° 26′ 28″ N 5° 13′ 22″ W) (Supplementary Fig. S7). In order to avoid similarity in *Acctinomycete* species, the soil samples were collected at five different points within a 400 m^2^ zone for each habitat^49^. For each point, the top five centimeters of soil was removed using a sterile spatula, and 150 to 200 g of soil from the underlying layer was collected, and placed in stomacher sachets, then mixed, and homogenized to generate a heterogeneous sample. The samples were transported aseptically to the laboratory, and conserved at 4 °C until use.

### Physico-chemical analysis of soil samples

The pH of each soil sample was determined using a pH meter (OCRISON, micro-pH 2000). Electrical conductivity (EC) was determined by a conductivity meter (sevenGoTM), and minerals including C, O, Mg, Si, Fe, K, and Ca were analyzed by scanning electron microscope (SEM) (JEOL: JSM-IT500HR) while other minerals (Zn, Mn, Cl, Al, P, Cu, and S) were analyzed by energy dispersive X-ray fluorescence method (Epsilon 3XLE from PANalytical, France). The organic matter (OM), and soil texture were analyzed according to the methods described by Jackson^73^.

### Pretreatment of soil samples, and isolation of *Actinomycetes* strains

In order to increase the number of *Actinomycetes*, the soil samples were pretreated using two methods: Drying method^49^, and the enrichment method^47^. Isolation was performed by the serial dilution method^74^. Four media were used to isolate *Actinomycetes*: (1) M2^31^, (2) GA^31^, (3) GLM^30,32^, and (4) Bennett^33^. Each medium was mixed after sterilization and cooling with 50 mg/L actidione (cycloheximide) to inhibit fungal growth. Plates (90 mm diameter) were incubated at 28 °C and monitored daily during three weeks to check for *Actinomycetes* growth. *Actinomycetes* colonies recognized by their macroscopic and microscopic aspects (Supplementary Fig. S8) were subcultured on ISP2 medium by the streak method in order to obtain pure cultures, and then were short-term stored in inclined tubes at 4 °C, and in 20% glycerol at − 20 °C for long-term storage^75^.

### Primary screening of *Actinomycetes* isolates for antimicrobial activity

In order to select the best medium for antibiotics production, the antimicrobial activity of *Actinomycetes* pure isolates was tested by the double-layer method^76^ using four Agar media (ISP1, ISP2, GYEA and Bennett) against different microorganisms: *Escherichia coli* ATCC 25,922, *Staphylococcus aureus* ATCC 25,923, *Bacillus ceureus* ATCC 14,579 (non pathogenic bacteria), *Candida albicans* ATCC 60,193 (pathogenic fungi) (these microoganisms were collected from the Institut Pasteur Casablanca Morocco), *Fusarium* sp. MN944566, *Fusarium* sp. MN944567*, Fusarium* sp. MN944568*, Fusarium* sp. MN944569*, Fusarium* sp. MN944570*, Fusarium* sp. MN944571*, Fusarium* sp. MN944572, *Fusarium* sp. MN944573*, Fusarium* sp. MN944574*, Fusarium* sp. MN944575*, Fusarium* sp*.* MN944576, and *Fusarium* sp*.* MN944577 (phytopathogenic fungi) (these phytopathogenic fungus were obtained from Laboratory of Agro-Alimentary and Health, Faculty of Sciences and Techniques, Hassan First University of Settat Moroco)*,* and 2 other fungi, *Trichoderma longibrachiatum* M37 and *Trichoderma koningii* M35, which cause invasive pulmonary and peritoneal infections respectively^77,78^. The antimicrobial activity was examined by measuring the zones of inhibition in mm. The phytopathogenic fungi (*Fusarium* sp.) were isolated from the mango tree *Mangifera indica* L. (infected at leaf level) native to the Indo-Burma region (senegal), is one of the oldest cultivated fruit trees in the world^79^.

### Production kinetics of secondary metabolites

The kinetics of secondary metabolite production was monitored during 10 days on ISP2 medium selected from the primary screening test for the five *Actinomycetes* isolates that exhibited high antimicrobial activity using the disc diffusion method^61,80^.

### Fermentation and extraction of secondary metabolites

The sixteen *Actinomycetes* isolates that showed significant antimicrobial activity in the primary screening were subjected to fermentation and subsequent extraction of secondary metabolites using organic solvents of increasing polarity. Indeed, Erlenmeyer flasks (500 mL) containing 100 mL of broth ISP2 culture medium chosen in the primary screening were inoculated with each active *Actinomycetes* isolate selected and incubated under constant agitation at 150 rpm under controlled conditions (28 °C).

In order to select the best solvent for extraction, the *Actinomycetes* cultures were centrifuged at 10,000 g for 20 min to remove the mycelial mass. One volume of supernatants was recovered and mixed vigorously in a separating funnel with the same volume of hexane. The aqueous phase obtained is then mixed with the same volume of dichloromethane and the same operations were done for the other organic solvents tested (ethyl acetate and butanol) by mixing in each time the aqueous phase obtained with the same volume of the next organic solvent tested. The organic extracts obtained were then evaporated at 45 °C. Finally the dry organic extracts obtained as well as the residual aqueous phases were solubilized in dimethylsulfoxide (DMSO) to calculate their concentrations^74,81–84^.

### Evaluation of the antimicrobial activity of organic extracts

The antimicrobial activity of the obtained organic extracts was evaluated by the disc diffusion method described by Badji et al.^76^ against six phytopathogenic fungi (*Fusarium* sp. MN944568*, Fusarium* sp. MN944569*, Fusarium* sp. MN944570*, Fusarium* sp. MN944571, *Fusarium* sp. MN9445677) and *Trichoderma longibrachiatum* M37 which induces invasive pulmonary infection^78^, and five clinical MDR strains (*Escherichia coli* 16D1150, *Enterococcus faecalis* 18K1386, *Staphylococcus aureus* 18K1052, *Proteus vulgaris* 16C1737, and *Neisseria gonorrhoeae* 16D1170). The antibiotic resistance profile of the clinical MDR strains tested was verified against sixteen antibiotics (Supplementary Table S7). Concerning antifungal activity, the tested fungi were subcultured on PDA medium and incubated at 28 °C for 10 days. A fungal suspension was obtained by adding a part of the tested isolate culture (mysilia and conedia) to sterile distilled water. The optical density of each inoculum was adjusted by a spectrophotometer set at a wave length of 623 nm in order to obtain an optical density between 0.18 and 0.20 which corresponds to about 10^6^ spore/mL^85^. A disc loaded with DMSO of a volume equal to that of the extract was used as a negative control. For the positive control, streptomycin and cycloheximide were used for antibacterial and antifungal activities, respectively. The diameter of the inhibition zones was measured after 24 h of incubation at 37 °C for bacteria and 48 h at 28 °C for fungi using foot caliper.

### Cultural, micro-morphological, biochemical and physiological characteristics of isolates

The cultural characteristics such as intensity of growth, pigmentation of medium, colony aspect, and the presence of diffusible pigments in agar were observed on ISP (ISP-1, ISP-2, ISP-4, ISP-5) and GYEA media^38^. All these media were seeded using striation method^38^ for each isolate, and then the plates (90 mm in diameter) were incubated at 28 °C and monitored daily during 10 days. The micro-morphological characters of pure isolates were determined under the optical microscope (Olympus CX43RF) in the fresh state and after Gram staining. Physiological and biochemical characters of *Actinomycetes* isolates tested were evaluated according to previous studies^17,81^. They concern the production of melanoid pigments, the tolerance to different increasing concentrations of sodium chloride (NaCl) and pH values, the growth at different degrees of temperature, and the assimilation of carbohydrates. Based on the cultural, biochemical and physiological characteristics, a hierarchical classification of *Actinomycetes* groups was performed using specific software (IBM SPSS statistics 23). After the analysis, a dendrogram was generated using the average distance among groups.

### Genomic DNA extraction, 16 rRNA gene sequencing and phylogenetic tree construction

The eleven *Streptomyces* sp. strains showed high antimicrobial activity were inoculated into 50 ml of ISP2 medium without Agar and subjected to constant agitation for 5–7 days until the turbidity of the medium was high. Cells were concentrated by centrifugation at 10,000 g for 20 min at 4 °C, and then congealed (without supernatant) in 2 mL eppendorfs tubes at − 20 °C until used^86^. DNA extraction was performed by an automated system (Mag Purix Bacterial DNA Extraction Kit) following the manufacturer's instructions. The DNA extracted from each isolate was quantified using a NanoDrop 8000 Spectrophotometer (thermo scientific) (Supplementary Fig. S9), and stocked at − 20 °C until use. All concentrations of extracted DNA samples were adjusted to100 ng/µL for the PCR reaction.

The 16S rRNA gene was amplified using the universal bacterial primer pairs Fd1 (5′-AGAGTTTGATCATGGCTCAG-3′) and rP2 (5′-ACGGTTACCTTGTTACGACTT-3′) to obtain an amplicon with a size of 1,500 bp with the reported conditions^87^. The amplified fragments were analyzed by electrophoresis. More precisely, 8 µL of PCR products were deposited on agarose gel (1%) in the presence of the 1 kb molecular weight marker (thermo scientific). The photo was visualized by the Gel-Documentation G-Box photo system (Supplementary Fig. S10).

The amplified products were purified using ExoSAP-IT PCR cleanup Kit (Applied Biosystems), and sequence reactions were performed by using a version 3.1 BigDye Terminator Cycle Sequencing Kit (Applied Biosystems). Sequencing products were purified using the BigDye® XTerminator™ Purification Kit (Applied Biosystems), and loaded onto an ABI 3130xL capillary sequencer (Applied Biosystems) according to the manufacturer’s instructions. The works of extraction, amplification and sequencing were carried out at the National Center of Scientific and Technical Research (CNRST, Rabat Morocco).

The assembly of the obtained sequences was performed using the version 5.15.0 DNA Baser Assembler software in order to generate the contigs, which have been recorded in FASTA form (.FASTA). Nucleotide sequences were aligned with MEGA X software^88^ using MUSCLE alignment. To designate the taxonomic status of the *Actinomycetes* isolates, the assembled and aligned sequences were compared with the database of non-redundant reference RNA sequences (refseq_rna) archived in the Genomic Data Bank, which is accessible on the internet at the National Center for Biotechnology Information (NCBI) (http://www.ncbi.nlm.nih.gov), using the nucleotide search program BLAST. The phylogenetic tree was constructed with the same alignment software using the neighbor-joining tree method^88^.

### Determination of total phenolic and flavonoid compounds

The determination of total phenolic compounds contained in the dichloromethane extracts of the five *Streptomyces* sp. strains showed high antimicrobial activity, was performed using the folin-ciocalteu method^89^. Gallic acid was used as standard. Quantification of flavonoid content in the same extracts was performed using the aluminum trichloride method^90^. Quercetin was used as standard.

### In vitro antioxidant activities

The free radical scavenging activity (DPPH) of the dichloromethane extracts of the five *Streptomyces* sp. strains showed high antimicrobial activity was performed according to the method described by Blois^91^. The absorbance was measured at 517 nm using an Elisa microplate reader (2100-C, Optic Ivymen Systems, Spain). Ascorbic acid was used as a positive control. Concerning the free radical scavenging activity (ABTS), it was performed according to the method developed by Re et al^92^. Trolox was used as a positive control.

### UV–visible analysis

The absorption spectra of the dichloromethane extracts of the five *Streptomyces* sp. strains showed high antimicrobial activity solubilized in DMSO were recorded between 190 and 850 nm using UV–vis scanning spectrophotometer (HACH Lange DR6000)^92^.

### GC–MS analysis of E23-4 strain crude dichloromethane extract

The volatile compound profile of the crude dichloromethane extract of E23-4 strain OM883990 was characterized by gas chromatography (GC) (Agilent 7890A Series) coupled to mass spectrometry (MS) equipped with a multimode injector and a HP-5MS column with a dimension of 30 m × 0.250 mm × 0.25 μm at the Moroccan Foundation for Advanced Science, Innovation and Research (MAScIR) Institute. Briefly, four µL of the extract solubilized in an adequate volume of chloroform was injected into the column by 1:4 split mode using helium as carrier gas at 1.7 mL/min. The temperatures of the ion source and quadrupoles were 230 and 150 °C, respectively. The oven temperature program was started at 60 °C and finished at 360 °C. The compound's identification was performed by comparing the obtained mass spectra with the data available in NIST 2017 MS Library^30,93^.

### Statistical analysis

All the experiments data were repeated three times and results were expressed as mean ± SD. Ordinary one-way ANOVA followed by Tukey's multiple comparisons test which was performed using GraphPad Prism 8.4.3 software (GraphPad software Inc., San Diego,CA,USA) to verify significant differences among groups in antioxidant activity tests and phenolic compound assays. Results were considered statistically significant when *P* ≤ 0.05. Pearson correlation analysis was performed using GraphPad Prism 8.4.3 software to determine the relationship between total *Actinomycetes* number and soil physico-chemical parameters as well as the relationship between phenolic compounds and antioxidant activity. Data measured in percentages were subjected to an arcsine transformation in order to approximate the normal distribution before analysis.

## Supplementary Information

Supplementary Information.

## Data Availability

All the data generated or analyzed during this study, including the nucleotide sequence data, are available as Supplementary Information files. Genome sequences of *Streptomyces* sp*.* E23-2, E23-3, E23-4, E23-8, E23-9, E23-10, E23-11, E24-9, E25-9, E25-11 and E25-12 have been deposited in National Centre for Biotechnology Information (NCBI) GenBank (https://www.ncbi.nlm.nih.gov/nucleotide/) under accession numbers of OM883988, OM883989, OM883990, OM883991, OM883992, OM883993, OM883994, OM883995, OM883996, OM883997 and OM883998.
