# Supplementary material for: Antimicrobial and antioxidant activities of Streptomyces species from soils of three different cold sites in the Fez-Meknes region Morocco
Source: Sci Rep. 2022 Oct 14;12:17233. doi: 10.1038/s41598-022-21644-z (PMC9568536; doi:10.1038/s41598-022-21644-z)
Supplement: Supplementary file 1 — Supplementary Information. [file 41598_2022_21644_MOESM1_ESM.pdf]

Antimicrobial and antioxidant activities of *Streptomyces* species from soils of three different cold sites in the Fez-Meknes region Morocco

Said Rammali[a,*], Lahoucine Hilali[a], Khadija Dari[a], Bouchaib Bencharki[a], Abdellatif Rahim[b], Mohammed Timinouni[c], Fatima Gaboune[d], Mohamed El Aalaoui[e], Abdelkrim khattabi[a]

[a]Laboratory of Agro-Alimentary and Health, Faculty of Sciences and Techniques, Hassan First University of Settat, B.P. 539, Settat 26000, Morocco

[b]Laboratory of Biochemistry, Neurosciences, Natural Ressources and Environment, Faculty of Sciences and Techniques, Hassan First University of Settat, B.P. 539, Settat 26000, Morocco

[c]Molecular Bacteriology Laboratory, Institut Pasteur du Maroc, Casablanca, Morocco

[d]National Institute of Agronomic Research of Rabat, Morocco

[e]Regional Center of Agronomic Research of Settat, Tertiary Road 1406, At 5 Km from Settat, Settat 26400, Morocco

* Corresponding authors:
s.rammali@uhp.ac.ma / rammali_fst@hotmail.fr (Said RAMMALI)

SUMMARY

1. General methods

1.1Recommended media for *Actinomycetes* isolation..3

1.2 Recommended media for testing antimicrobial activity...3

1.3 Recommended medium for testing NaCl tolerance..3

1.4 Recommended medium for the production of melanin pigment.......................................4

1.5 Other media used in this study..4

2. Supplementary figures

Figure S1. Analysis of minerals using SEM...5

Figure S2. Production kinetics of antibiotics against *Bacillus cereus* ATCC 14579......................6

Figure S3. Microscopic aspect of the most active *Streptomyces* (Gram stain)................................7

Figure S4. Morphological aspect of the colonies of five isolates of active *Streptomyces*...............8

Figure S5. GC-MS of dichloromethane extract of E23-4 OM883990 strain with peaks indicating the presence of bioactive compounds (according to the NIST database).......................................9

Figure S6. Chemical structures of 21 secondary metabolites present in dichloromethane extract of E23-4 OM883990 strain analyzed by GC-MS..14

Figure S7. A map of the sampling sites retrieved from Google Maps (Google Maps. Map Data ©2022. Retrieved from https :(https://www.google.com/maps/@32.9937363,-7.6005892,15z)..... 15

Figure S8. *Actinomycetes* isolates obtained in the three sites (A, B, and C) according to their culture media...16

Figure S9. Determination of the extracted DNA by Nanodrop 8000..17

Figure S10. Agarose gel electrophoresis for amplification of genes coding for 16S rRNA of *Actinomycetes* strains...19

Figure S11. Chemical structures of secondary metabolites present in DM E23-4 extract of *Streptomyces* sp. strain analyzed by GC-MS...20

3. Supplementary tables

Supplementary Table 1. Morphological characteristics of active *Streptomyces* isolates..............21

Supplementary Table 2. Molecular identification of the 11 selected *Actinomycetes* isolates based on 16S rRNA gene sequencing..22

Supplementary Table 3. Spearman correlation between soil physico-chemical parameters and total number of *Streptomyces* (TNS) in the three sites A, B and C..23

Supplementary Table 4. Resistance profile of multi-drug resistant clinical bacteria...................24

1. General methods

1.1 Recommended media for *Actinomycetes* isolation

M2: 10 g of starch, 0.3 g of casein, 2 g of KNO_3,2 g of NaCl, 0.05 g of $MgSO_4$,$7H_2O$, 2 g of K_2HPO_4,0.02 g of $CaCO_3$, 0.01 g of $FeSO_4$, $7H_2O$, 1 g of glucose, 15 g of agar, 1L distilled water, and pH adjusted at 7. 2[1].

GA: 10 g of Glucose, 0.5 g of asparagine, 0.5 g of K_2HPO_4, 15 g of agar, 1L distilled water, and pH adjusted at 6[1].

GLM: 10 g of glucose, 3 g of yeast extract, 3 g of malate extract, 5 g of tryptone, 15 g of agar, 1L distilled water, and pH adjusted at 7.2[2,3].

Bennett : 10 g of glucose, 2 g of yeast extract, 1 g of meat extract, 2 g of tryptone, 15 g of agar, 1L distilled water, and pH adjusted at 7.2[4].

1.2 Recommended media for testing antimicrobial activity[5]

ISP1: 3 g of yeast extract, 5 g of tryptone, 16 g of agar, 1L of distilled water, and pH adjusted at 7.15.

ISP2: 4 g of yeast extract, 4 g of glucose, 10 g of malate extract, 16 g of agar, 1L of distilled water, and pH adjusted at 6.51.

GYEA: 10 g of yeast extract, 10 g of glucose, 16 g of agar, 1L of distilled water, and pH adjusted at 6.96.

Bennett: 10 g of glucose, 2 g of yeast extract, 1 g of meat extract, 2 g of tryptone, 15 g of agar, 1L distilled water, and pH adjusted at 7.2.

1.3 Recommended medium for testing NaCl tolerance

YEA medium: 3 g of yeast extract, 5 g of peptone, 15 g of agar, 1L of distilled water, and pH adjusted at 7.2.

1.4 Recommended medium for the production of melanoid pigment

ISP9: 2.64 g of (NH_2) SO_4, 2.38 g of KH_2PO_4, 5.65 g of K_2HPO_4, 1 g of $MgSO_4$, $7H_2O$, 20 g of agar, and 1L of distilled water.

100 mL salt solution: 4 g of $CuSO_4$, $5H_2O$, 1.1 g of $FeSO_4$, $7H_2O$, 7.9 g of $MnCl_2$, $4H_2O$, 1.5 g of $ZnSO_4$, 7H2O.

1.5 Other media used in this study

PDA medium: 4 g of potato extract 20 g of glucose, 15 g of agar, 1L distilled water, and pH adjusted at 7.5.

Muller Hinton Agar medium: Ready to use

CLED medium: Ready to use.

2. Supplementary figures

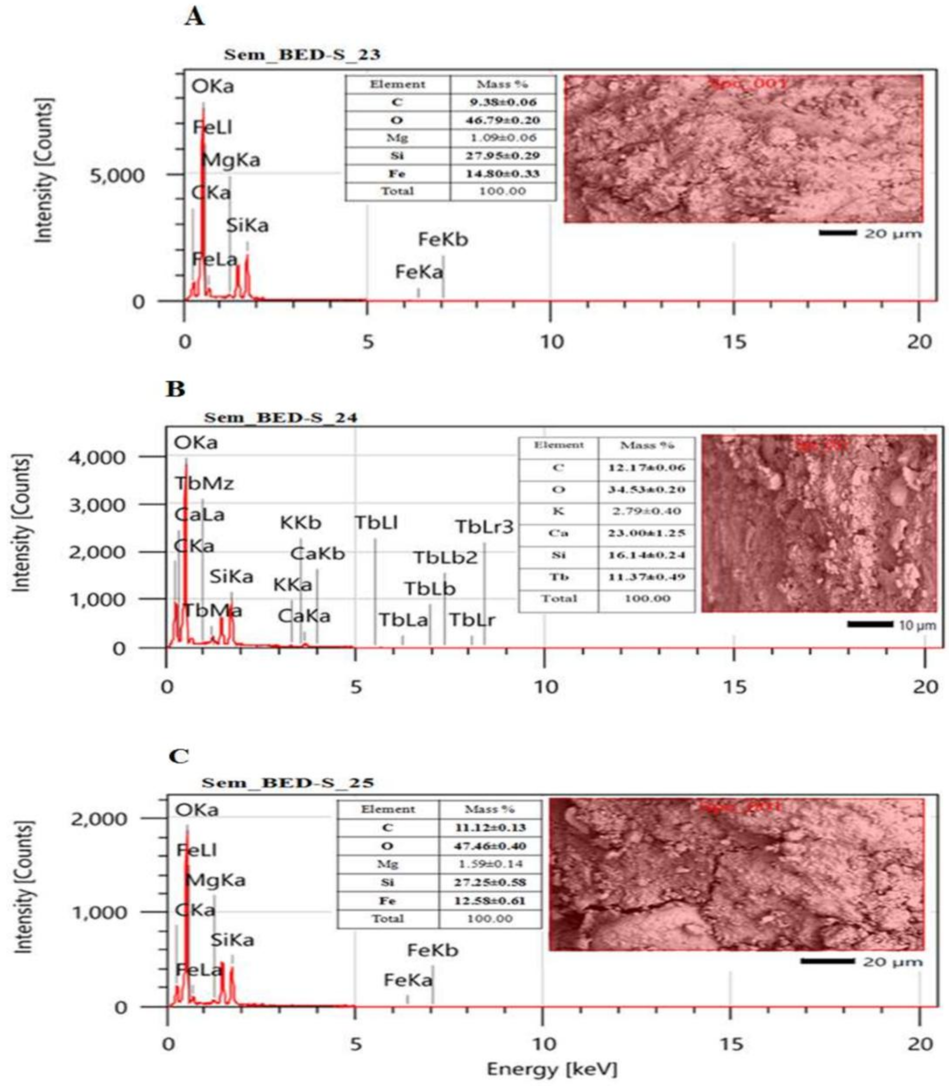

Figure S1. Analysis of minerals using scanning electron microscope (SEM).

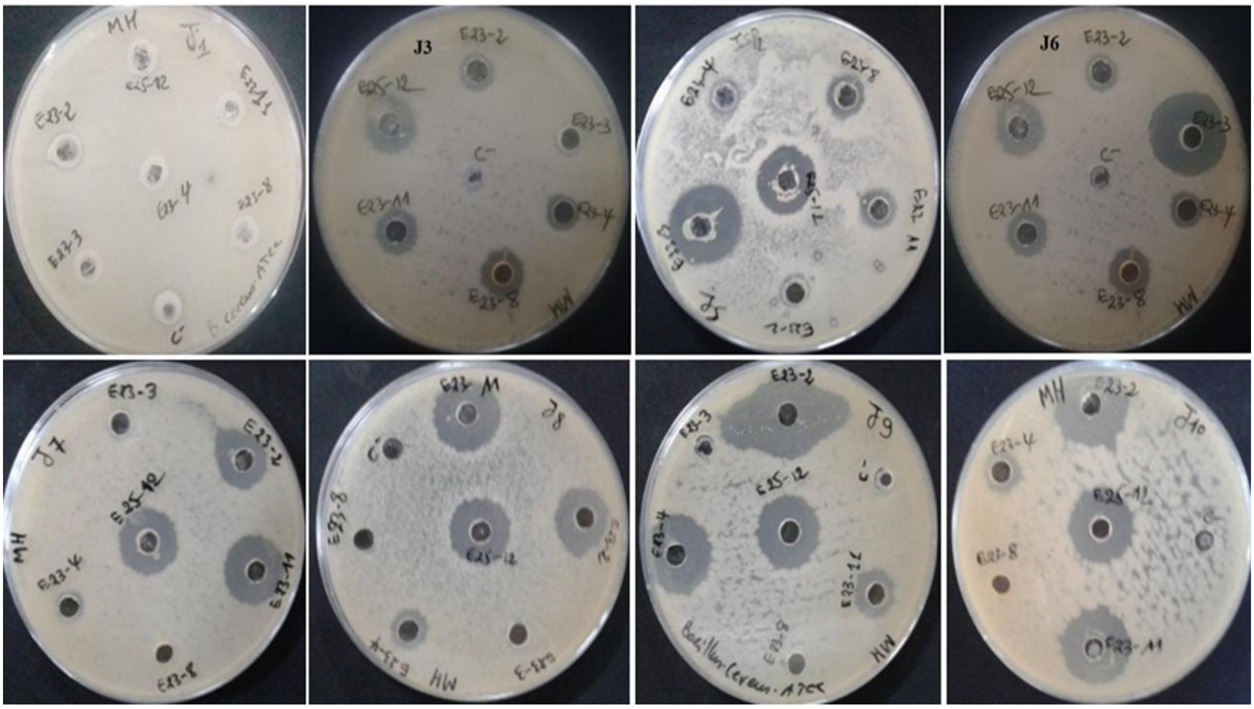

Figure S2. Production kinetics of antibiotics against *Bacillus cereus* ATCC 14579.

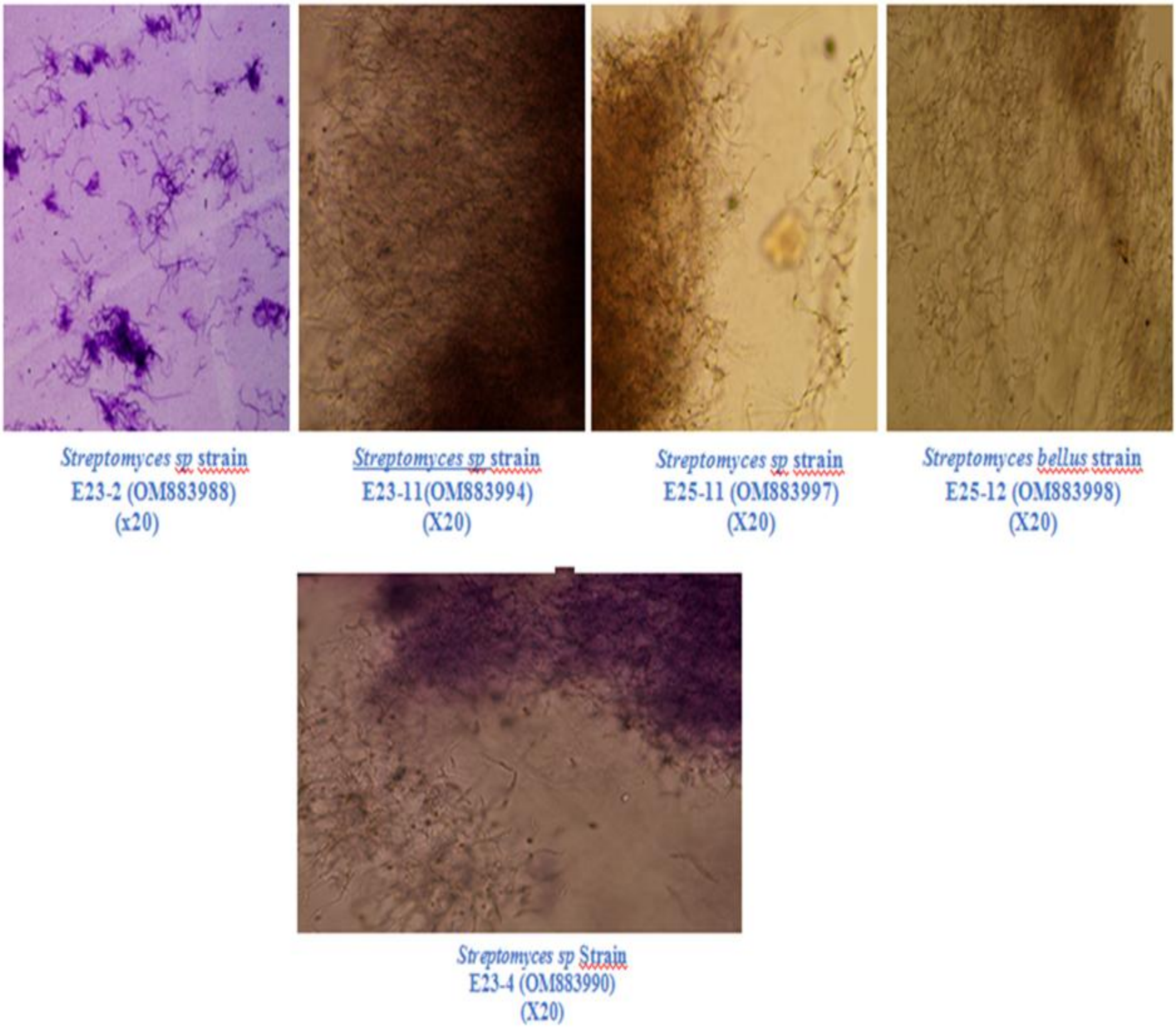

Figure S3. Microscopic aspect of the most active *Streptomyces* (Gram staining).

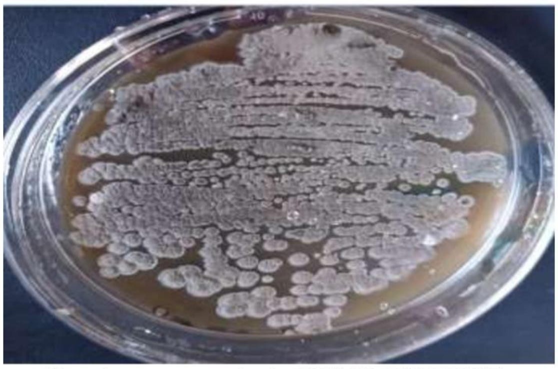

Streptomyces sp. strain E25-11 OM883997

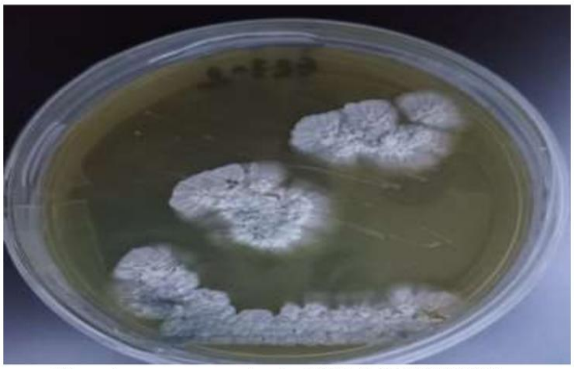

Streptomyces sp. strain E23-2 OM883988

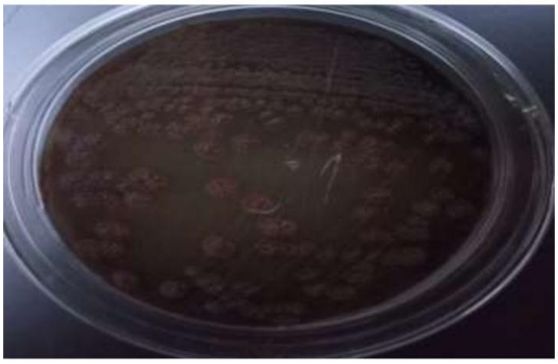

Streptomyces sp. strain E23-4 OM883990

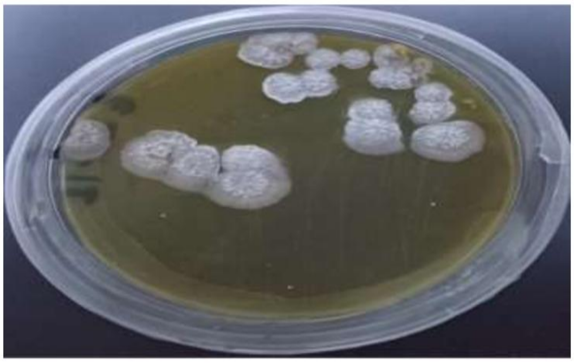

Streptomyces bellus strain E25-12 OM883998

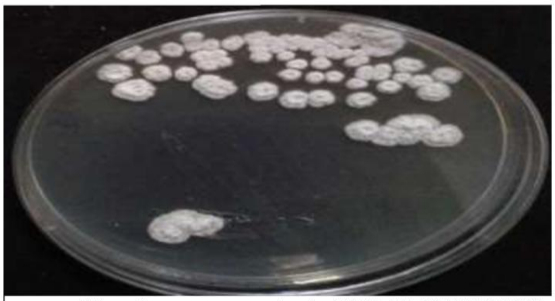

Streptomyces sp. strain E23-11 OM883994

Figure S4. Morphological aspect of the colonies of five isolates of active *Streptomyces*

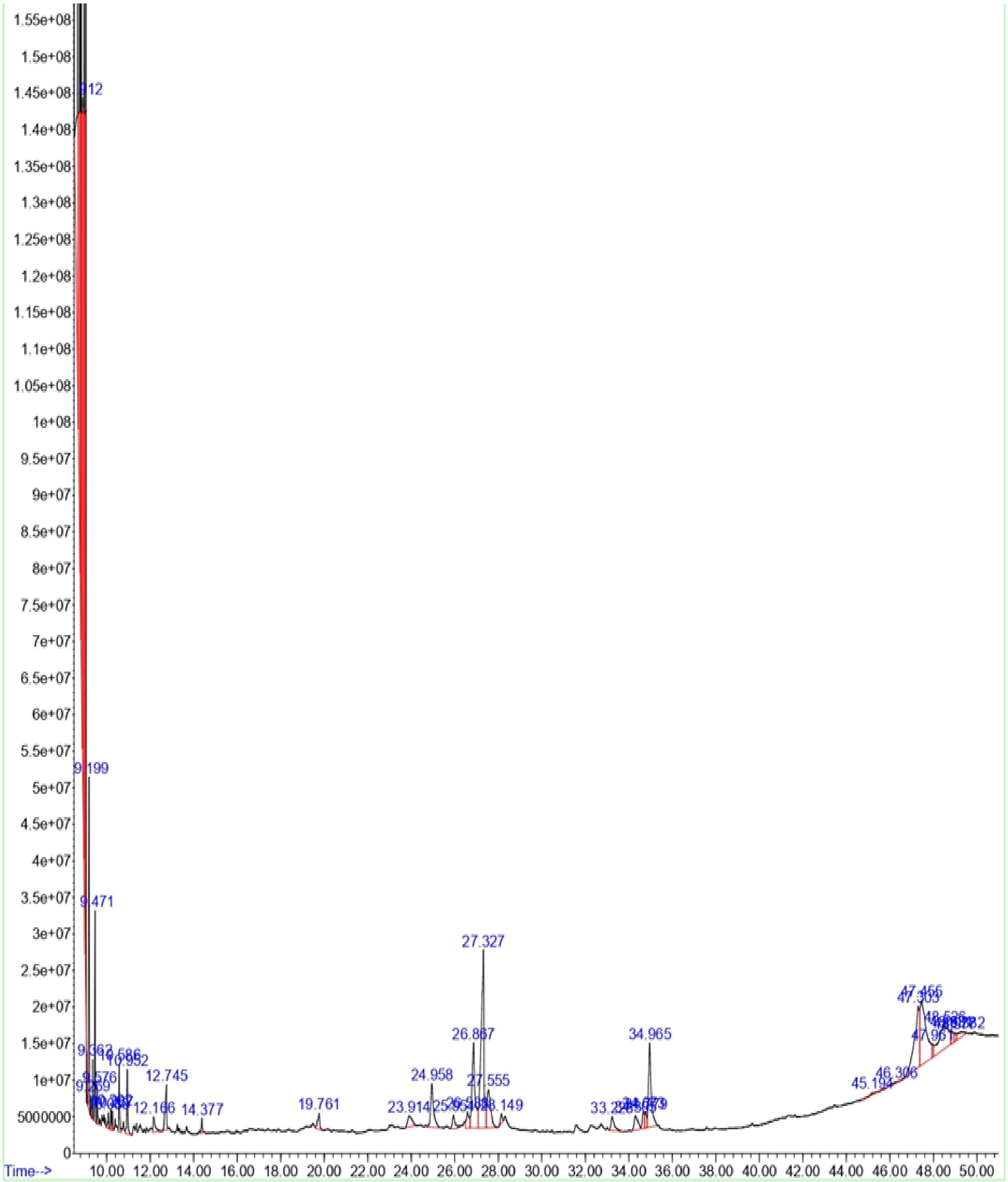

Figure S5. GC-MS of dichloromethane crude extract of E23-4 OM883990 strain with peaks indicating the presence of bioactive compounds (according to the NIST database).

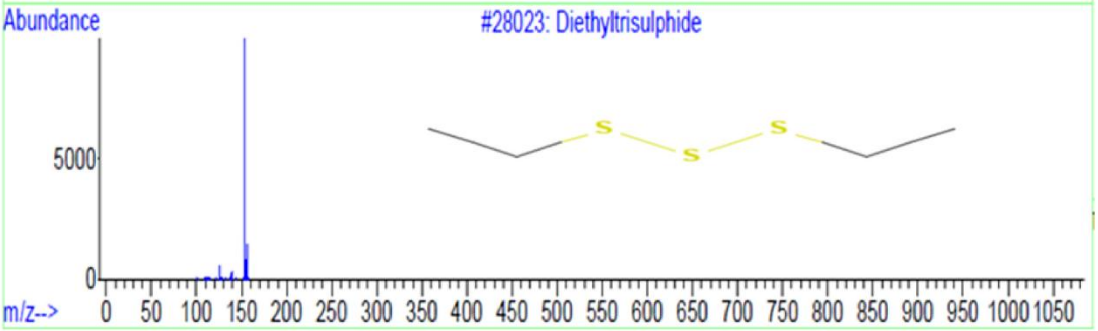
Abundance
#28023: Diethyltrisulphide
5000
0
m/z-->
0 50 100 150 200 250 300 350 400 450 500 550 600 650 700 750 800 850 900 950 1000 1050
S
S
S

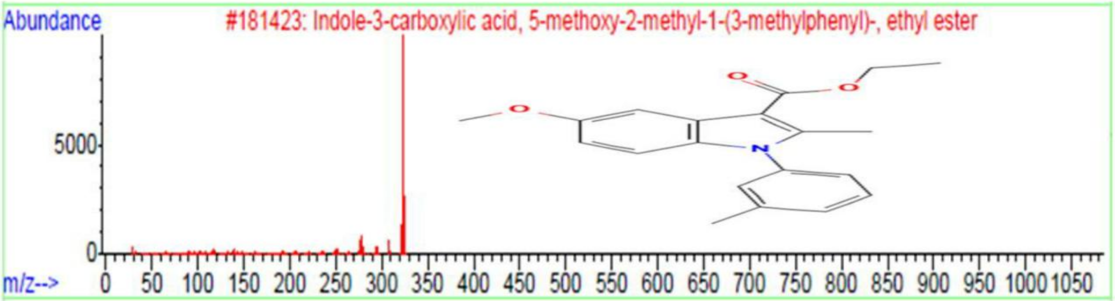
Abundance
#181423: Indole-3-carboxylic acid, 5-methoxy-2-methyl-1-(3-methylphenyl)-, ethyl ester
5000
0
m/z-->
0 50 100 150 200 250 300 350 400 450 500 550 600 650 700 750 800 850 900 950 1000 1050
O
O
O
N

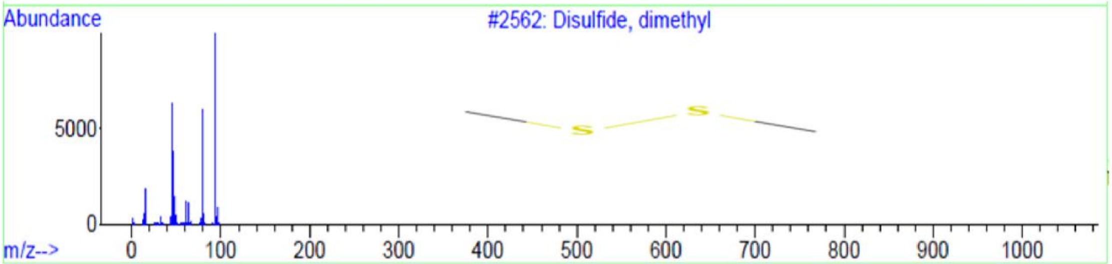
Abundance
#2562: Disulfide, dimethyl
5000
0
m/z-->
0 100 200 300 400 500 600 700 800 900 1000
S
S

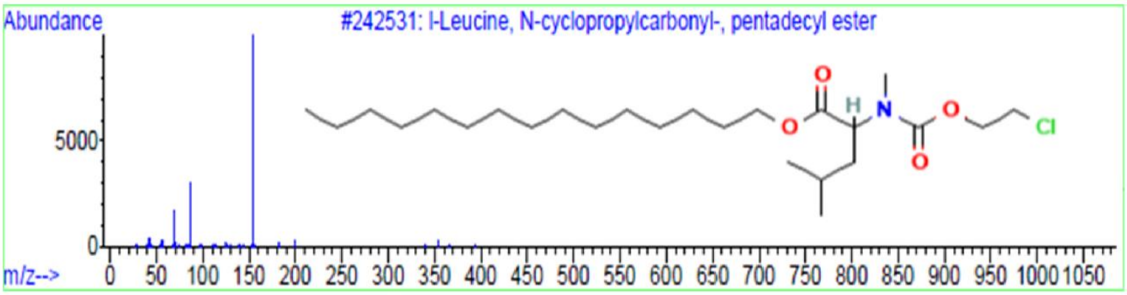
Abundance
#242531: l-Leucine, N-cyclopropylcarbonyl-, pentadecyl ester
5000
0
m/z-->
0 50 100 150 200 250 300 350 400 450 500 550 600 650 700 750 800 850 900 950 1000 1050
O
O
H
N
O
O
Cl

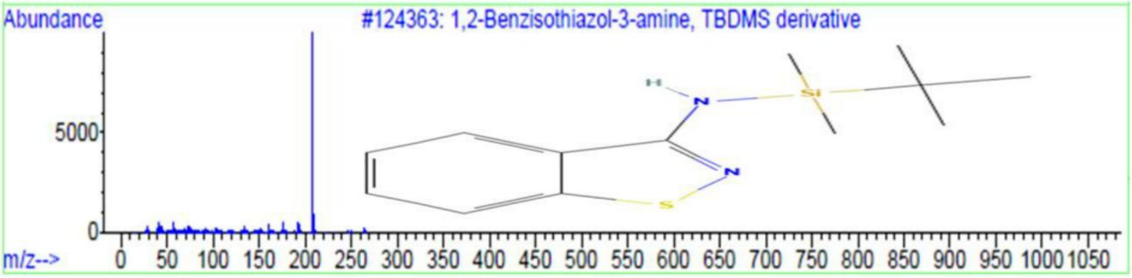
Abundance
#124363: 1,2-Benzisothiazol-3-amine, TBDMS derivative
5000
0
m/z-->
0 50 100 150 200 250 300 350 400 450 500 550 600 650 700 750 800 850 900 950 1000 1050
H
N
Si
N
S

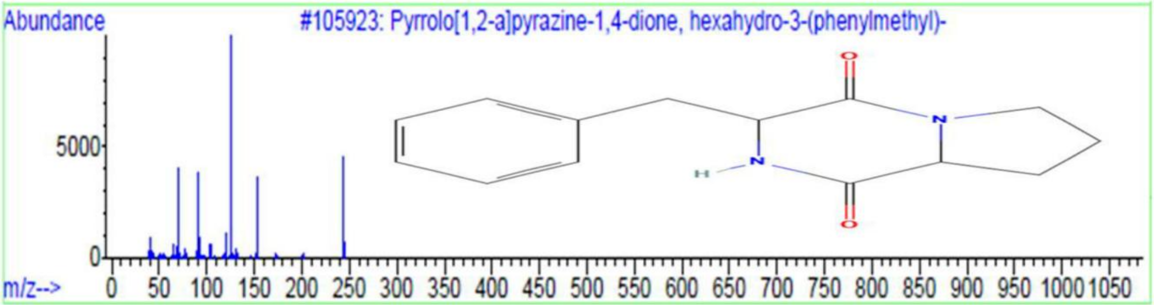
Abundance
#105923: Pyrrolo[1,2-a]pyrazine-1,4-dione, hexahydro-3-(phenylmethyl)-
5000
0
m/z-->
0 50 100 150 200 250 300 350 400 450 500 550 600 650 700 750 800 850 900 950 1000 1050

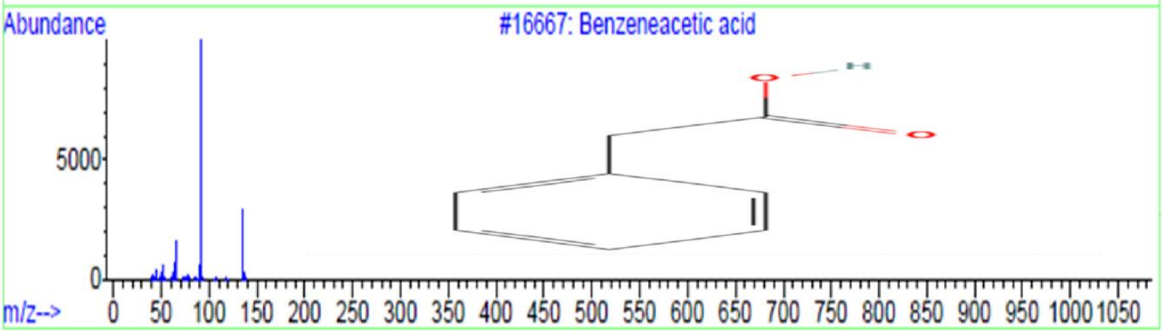
Abundance
#16667: Benzeneacetic acid
5000
0
m/z-->
0 50 100 150 200 250 300 350 400 450 500 550 600 650 700 750 800 850 900 950 1000 1050

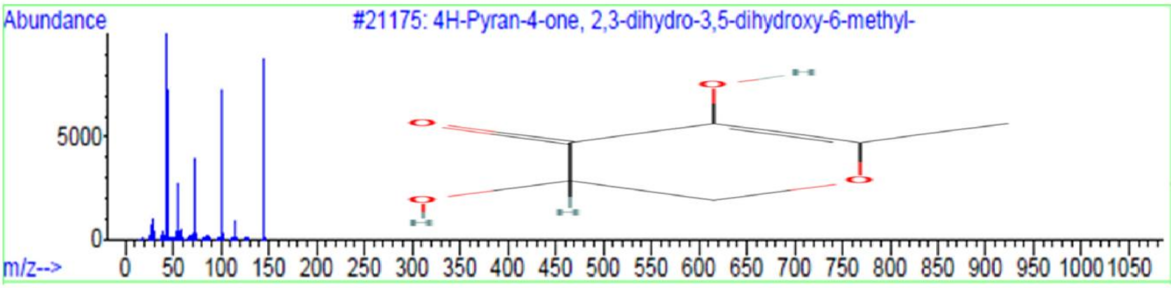
Abundance
#21175: 4H-Pyran-4-one, 2,3-dihydro-3,5-dihydroxy-6-methyl-
5000
0
m/z-->
0 50 100 150 200 250 300 350 400 450 500 550 600 650 700 750 800 850 900 950 1000 1050

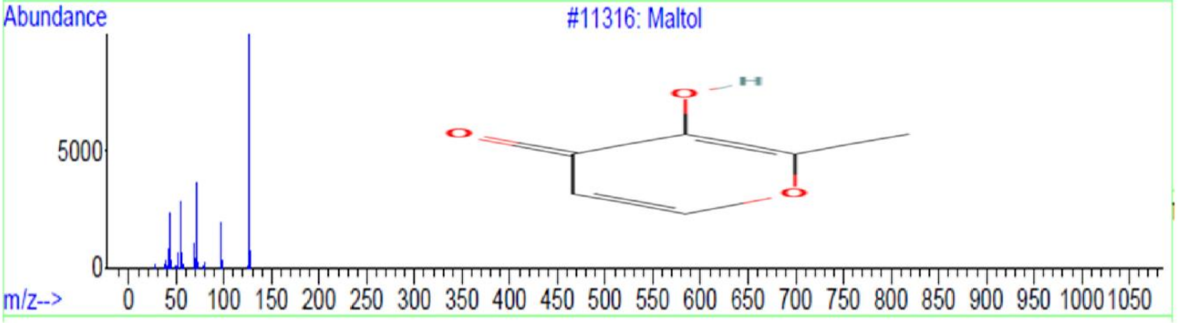
Abundance
#11316: Maltol
5000
0
m/z-->
0 50 100 150 200 250 300 350 400 450 500 550 600 650 700 750 800 850 900 950 1000 1050

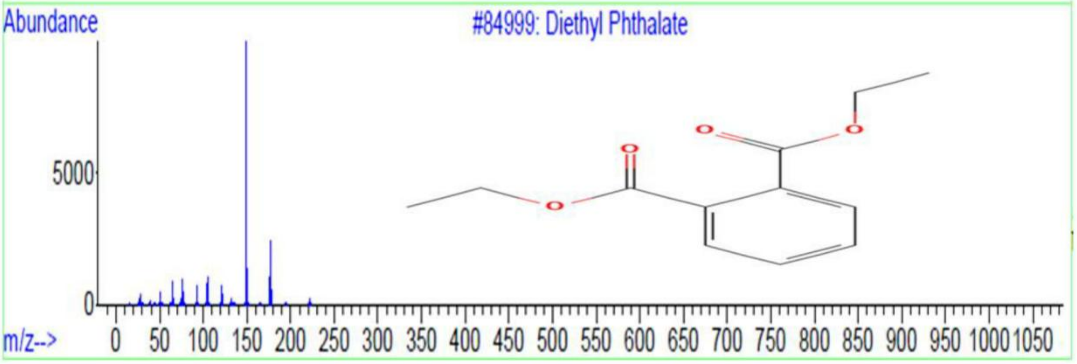
Abundance
#84999: Diethyl Phthalate
5000
0
m/z-->
0 50 100 150 200 250 300 350 400 450 500 550 600 650 700 750 800 850 900 950 1000 1050

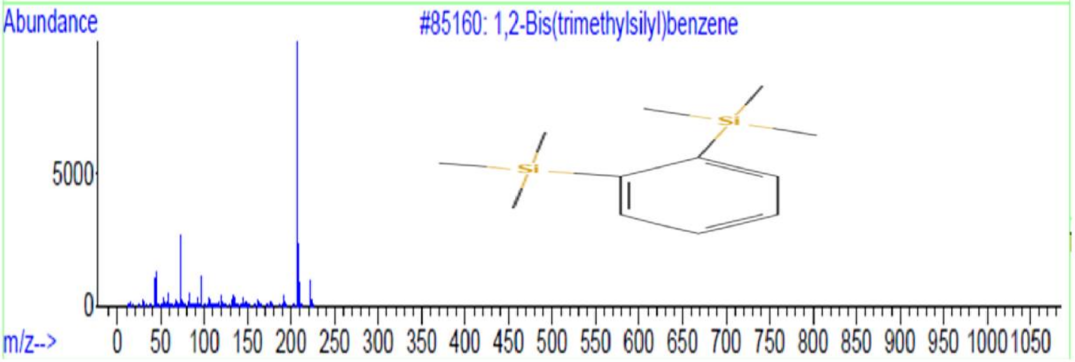
Abundance
#85160: 1,2-Bis(trimethylsilyl)benzene
Si
Si
5000
0
m/z-->
0 50 100 150 200 250 300 350 400 450 500 550 600 650 700 750 800 850 900 950 1000 1050

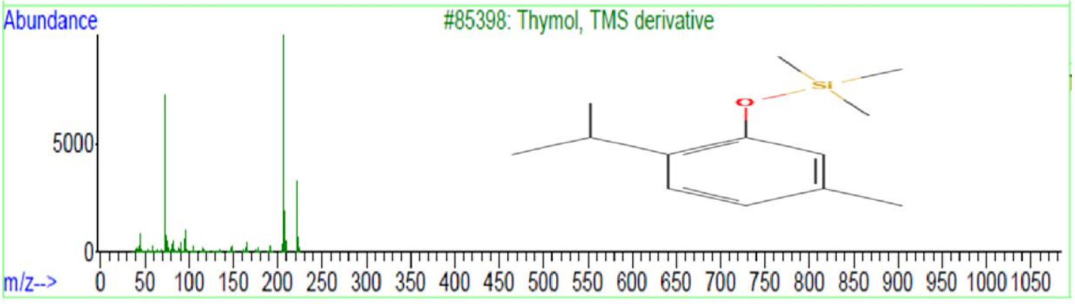
Abundance
#85398: Thymol, TMS derivative
Si
O
5000
0
m/z-->
0 50 100 150 200 250 300 350 400 450 500 550 600 650 700 750 800 850 900 950 1000 1050

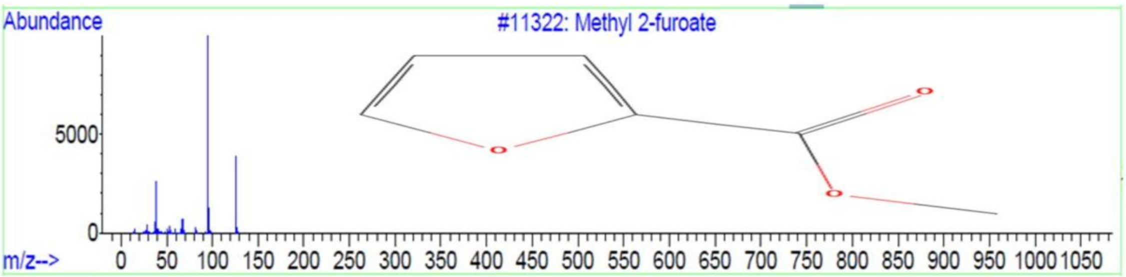
Abundance
#11322: Methyl 2-furoate
5000
0
m/z-->
0 50 100 150 200 250 300 350 400 450 500 550 600 650 700 750 800 850 900 950 1000 1050
O
O
O

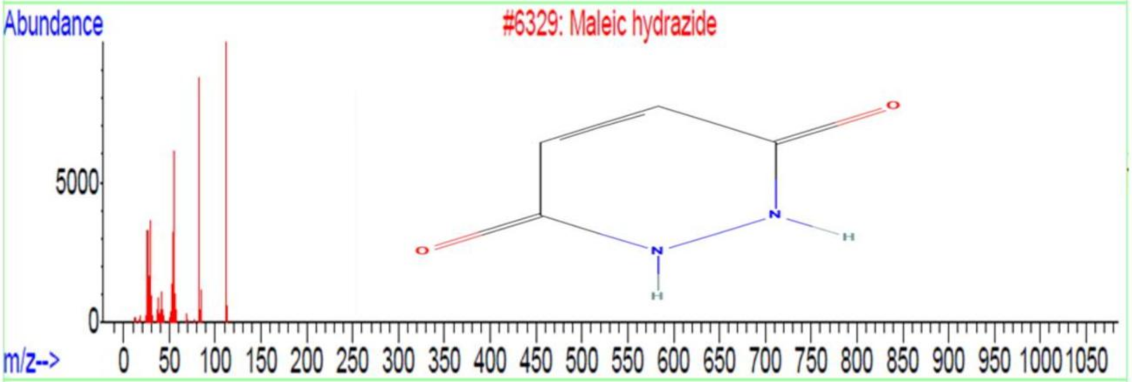
Abundance
#6329: Maleic hydrazide
5000
0
m/z-->
0 50 100 150 200 250 300 350 400 450 500 550 600 650 700 750 800 850 900 950 1000 1050
O
N
H
O
N
H

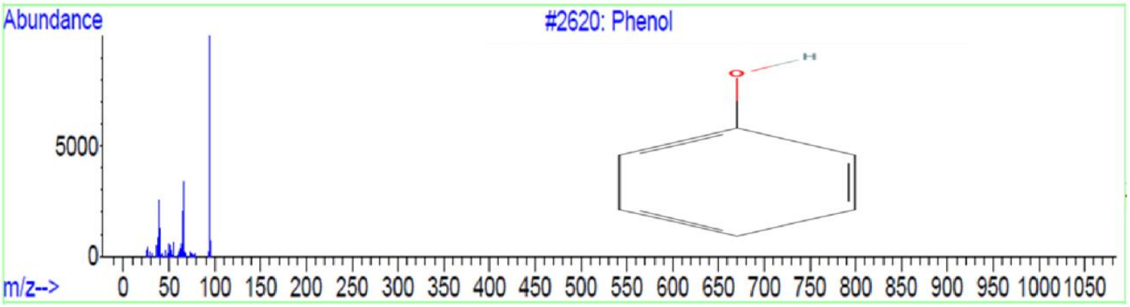
Abundance
#2620: Phenol
5000
0
m/z-->
0 50 100 150 200 250 300 350 400 450 500 550 600 650 700 750 800 850 900 950 1000 1050
O
H

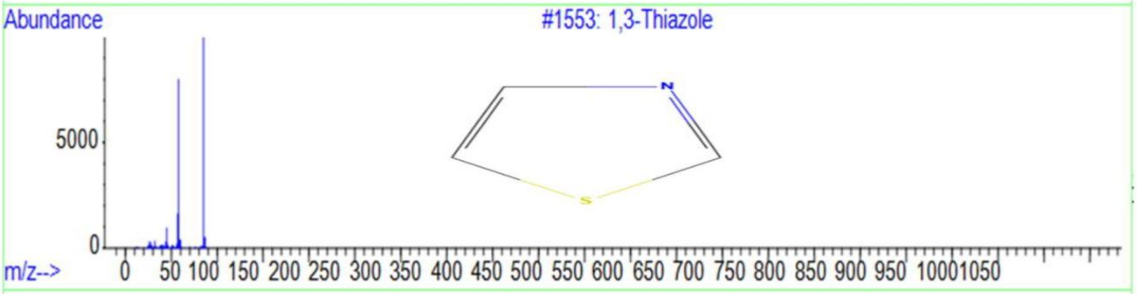
Abundance
#1553: 1,3-Thiazole
5000
0
m/z-->
0 50 100 150 200 250 300 350 400 450 500 550 600 650 700 750 800 850 900 950 1000 1050
N
S

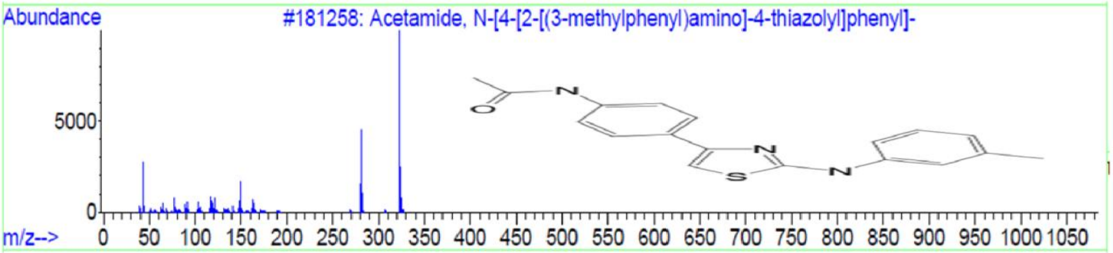

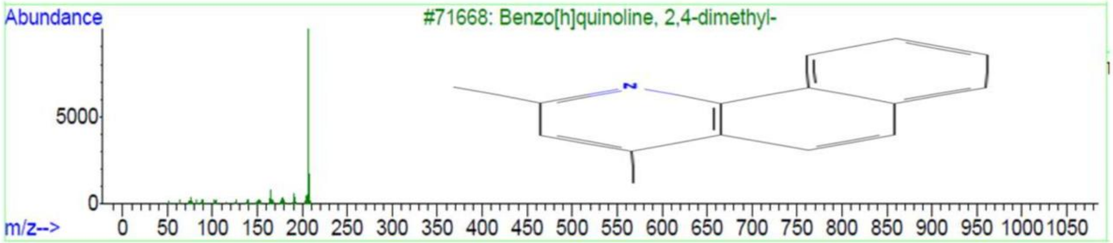

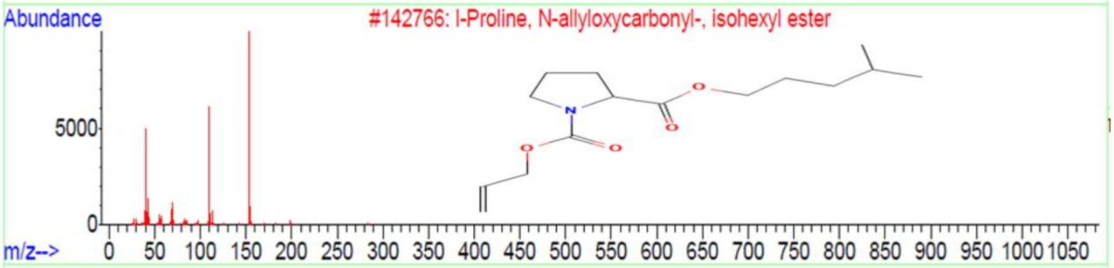

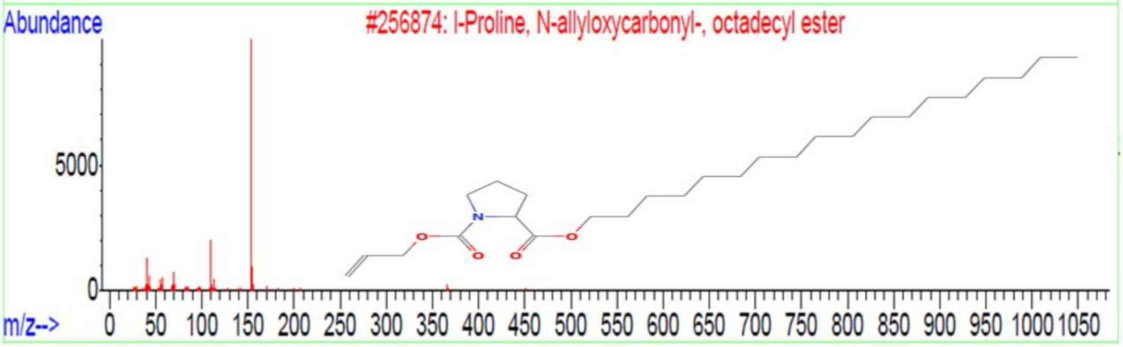

Figure S6. Chemical structures of 21 secondary metabolites present in dichloromethane extract of E23-4 OM883990 strain analyzed by GC-MS.

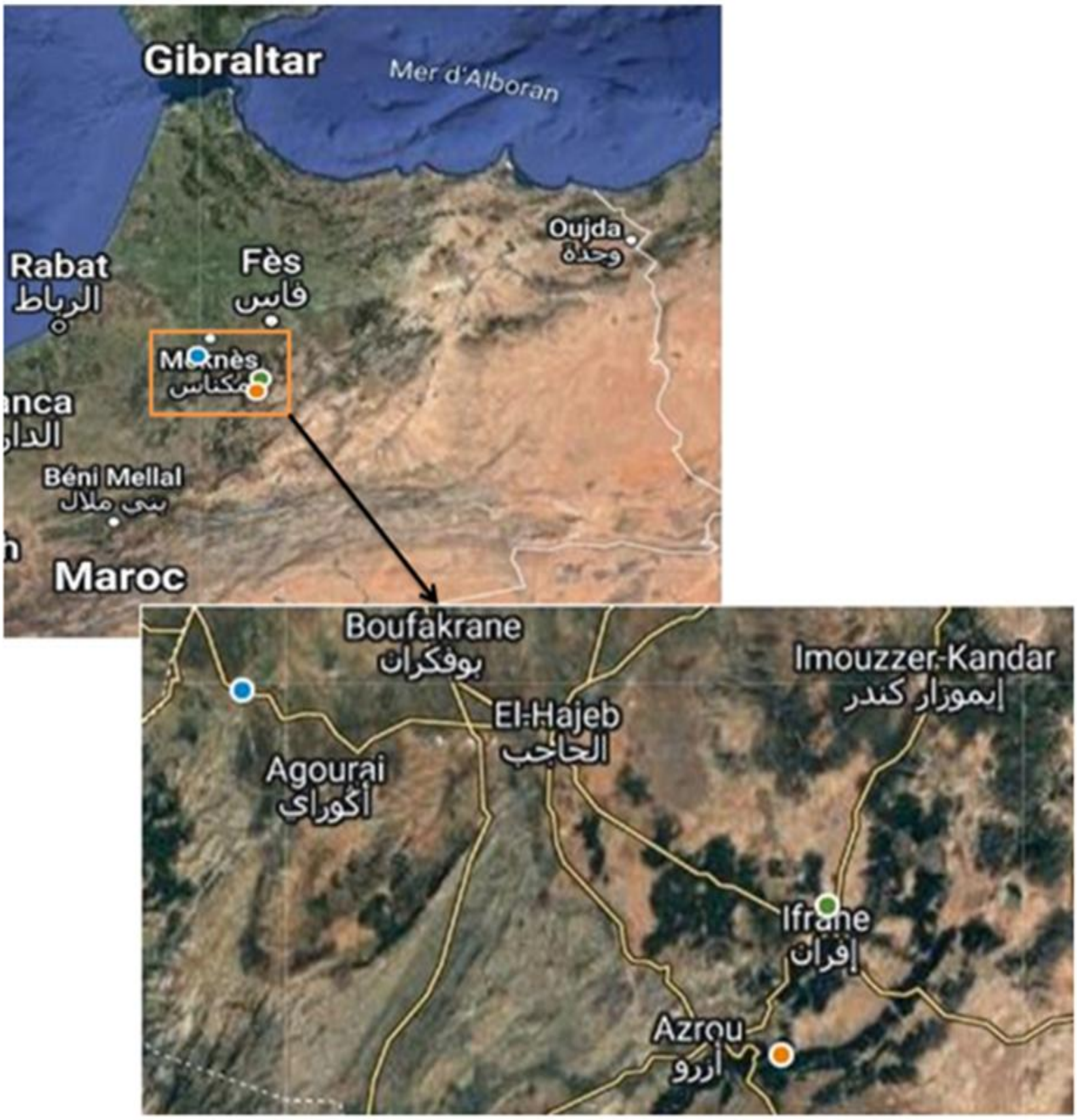

Figure S7. A map of the sampling sites retrieved from Google Maps (Google Maps. Map Data ©2022. Retrieved from https: https://www.google.com/maps/@32.9937363,-7.6005892,15z).

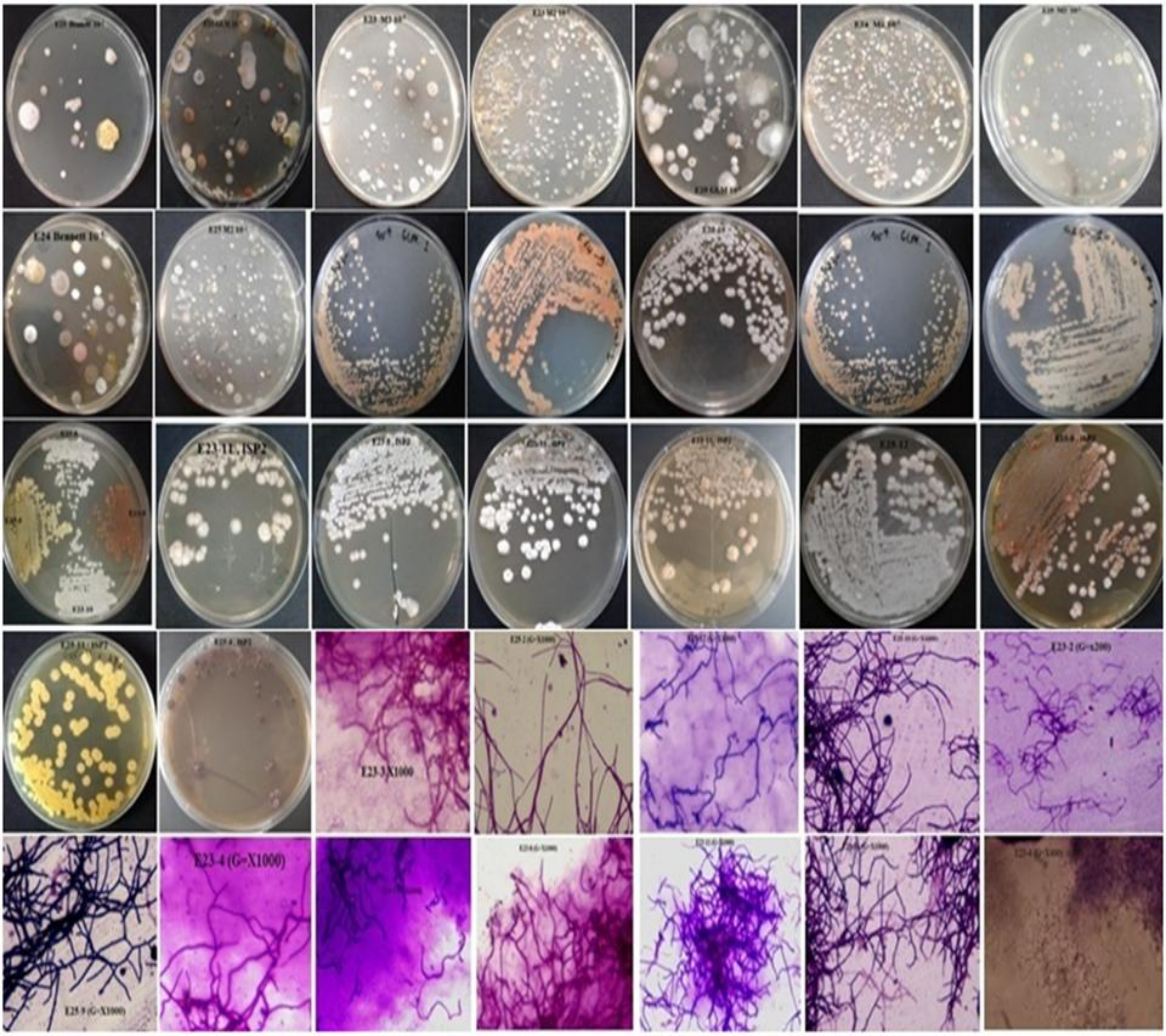

Figure S8. ***Actinomycetes*** **isolates obtained in the three studied (A, B and C) according to the culture media used.**

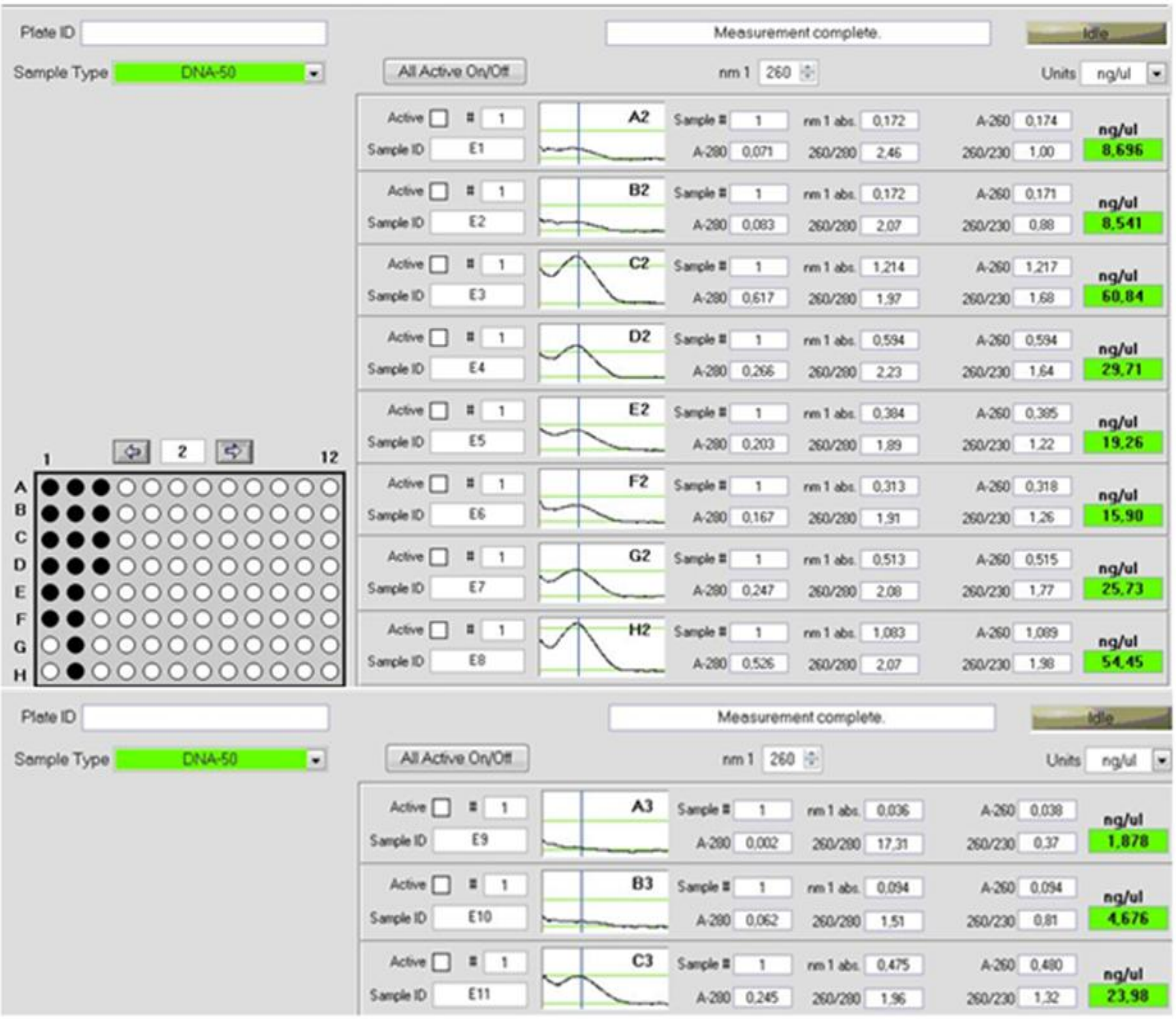

Figure S9. Determination of the extracted DNA by Nanodrop 8000.

The concentrations of the 11 samples are showed in the figure below.

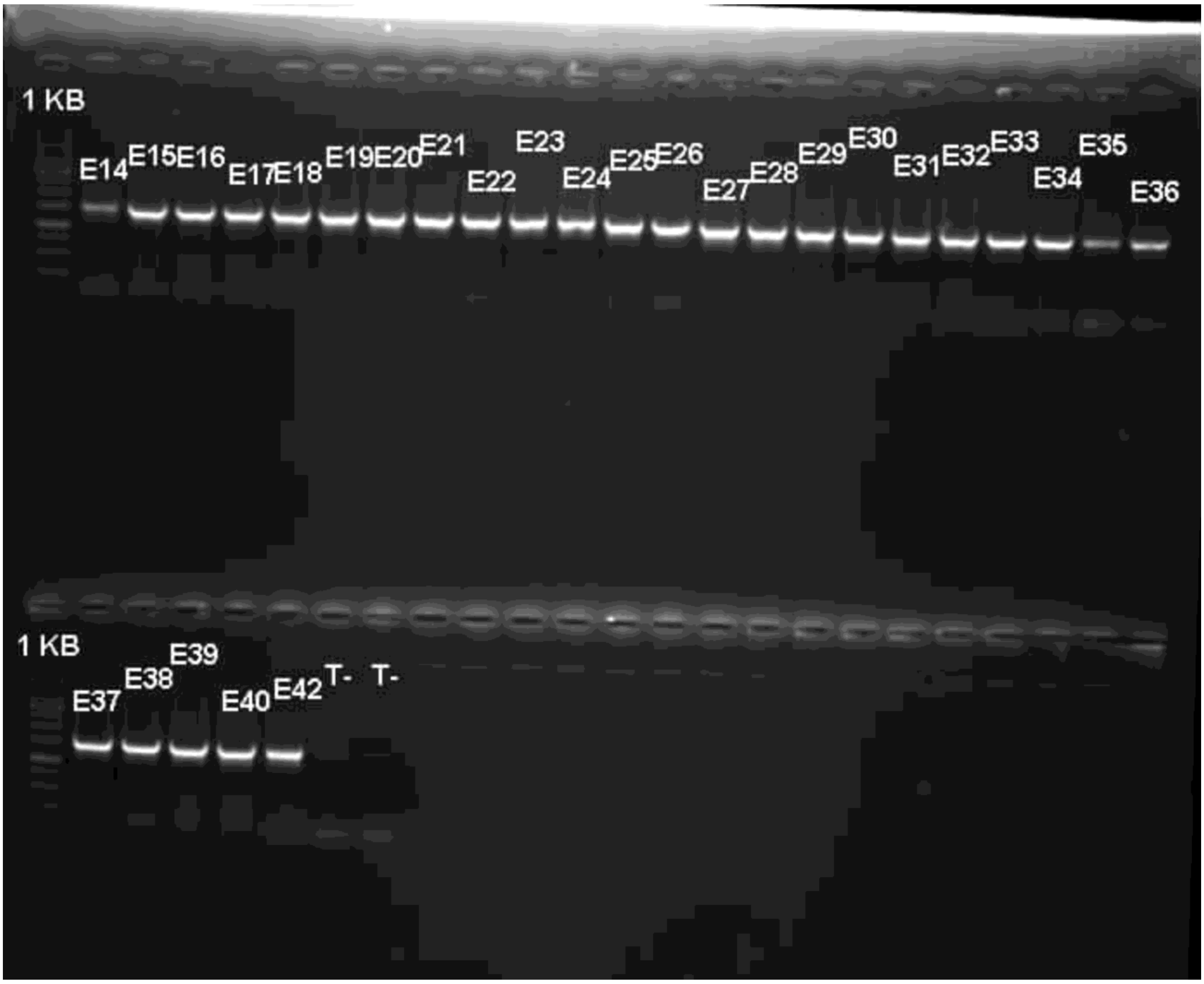

Figure S10. Agarose gel electrophoresis for amplification of genes coding for 16S rRNAof *Actinomycetes* strains.

Bands were fractionated by electrophoresis on a agarose gel (1 %) and visualised by the photo documentation system " G Box ".

3. Supplementary tables

Supplementary Table 1: Morphological characteristics of active *Streptomyces* isolates.

Strains	Identification (Scientific Name)	NCBI GenBank accession number	Colony aspect	Surface (aerial mycelium)	Reverse (substrate mycelium)	Pigmentation of the medium	Gram staining
E23-2	*Streptomyces* sp.	OM883988	Circular	Whitish, not powdery	yellowish, Orange	Absent	Gram-positive filaments
E23-3	*Streptomyces africans*	OM883989	Starry	Whitish, not powdery	yellowish	Absent	Gram-positive filaments
E23-4	*Streptomyces* sp.	OM883990	Circular	Brick	Brick	Brownish	Gram-positive filaments
E23-8	*Streptomyces* sp.	OM883991	Circular	Whitish, not powdery	yellowish, pale	Absent	Gram-positive filaments
E23-9	*Streptomyces galilaeus*	OM883992	Circular, Starry	Orange	Orange	Absent	Gram-positive filaments
E23-10	*Streptomyces bellus*	OM883993	Punctiform	Whitish, not powdery	yellowish, Orange	Absent	Gram-positive filaments
E23-11	*Streptomyces* sp.	OM883994	Circular, Starry	Beige	yellowish, pale	Absent	Gram-positive filaments
E24-9	*Streptomyces amritsarensis*	OM883995	Circular	Pinkish	Orange	Absent	Gram-positive filaments
E25-9	*Streptomyces* sp.	OM883996	Starry	Beige	yellowish, Orange	Absent	Gram-positive filaments
E25-11	*Streptomyces* sp.	OM883997	Starry	Beige	yellowish, Orange	Absent	Gram-positive filaments
E25-12	*Streptomyces bellus*	OM883998	Punctiform	Whitish, powdery	yellowish, Orange	Absent	Gram-positive filaments

Supplementary Table 2: Molecular identification of the 11 selected *Actinomycetes* isolates based on 16S rRNA gene sequencing.

Isolate Name	Query Length (bp)	Nearest known species[a]	Max Score	Query Coverage (%)	E-value	Percent identity (%)	Identification[b] (Scientific Name)	NCBI GenBank accession number(s)
E23-2	1393	*Streptomyces bellus* NBRC 12844(T) (NR_041222.1) /*Streptomyces coerulescens* CSSP046(T) (NR_043337.1)	2477	100	0.0	98.92	*Streptomyces* sp.	OM883988
E23-3	1388	*Streptomyces africanus* NBRC 101005 (T) (NR_112600.1)	2490	100	0.0	99.06	*Streptomyces africans*	OM883989
E23-4	1399	*Streptomyces novaecaesareae* NBRC 13368 (T) (NR_041124.1)/ *Streptomyces resistomycificus* NBRC 12814 (T) (NR_112287.1) / *Streptomyces pseudovenezuelae* NBRC 12904 (T) (NR_041090.1)	2473	100	0.0	98.65	*Streptomyces* sp.	OM883990
E23-8	1402	*Streptomyces coeruleofuscus* CSSP429 (T) (NR_115381.1) / *Streptomyces bellus* NBRC 12844 (T) (NR_041222.1)	2431	100	0.0	98.15	*Streptomyces* sp.	OM883991
E23-9	1395	*Streptomyces galilaeus* NBRC 13400 (T) (NR_112389.1)/ *Streptomyces galilaeus* JCM 4757 (T) (NR_040857.1)	2516	100	0.0	99.28	*Streptomyces galilaeus*	OM883992
E23-10	1231	*Streptomyces bellus* NBRC 12844(T) (NR_041222.1)	2246	100	0.0	99.59	*Streptomyces bellus*	OM883993
E23-11	1508	*Streptomyces bellus* NBRC 12844 (T) (NR_041222.1)/*Streptomyces coeruleofuscus* CSSP429 (T) (NR_115381.1)	2425	94	0.0	97.56	*Streptomyces* sp.	OM883994

E24-9	1373	*Streptomyces amritsarensis* 2A (T) (NR_126204.1)	2497	99	0.0	99.63	*Streptomyces amritsarensis*	OM883995
E25-9	1416	*Streptomyces afghaniensis*NBRC 12831 (T) (NR_041221.1) /*Streptomyces africanus* NBRC 101005 (T) (NR_112600.1)	2377	100	0.0	97.25	*Streptomyces* sp.	OM883996
E25-11	1576	*Streptomyces avermitilis* (T) (NR_074747.2) /*Streptomyces avermitilis* NBRC 14893 (T) (NR_112447.1)	2006	94	0.0	91.61	*Streptomyces* sp.	OM883997
E25-12	1394	*Streptomyces bellus* NBRC 12844 (T) (NR_041222.1)	2545	100	0.0	99.64	*Streptomyces bellus*	OM883998

[a]NCBI's nucleotide BLAST program was used to find the closest match against the non-redundant reference RNA sequence database (refseqrna). The strain number, strain type (T), and GenBank accession number are all listed after the species name.

[b]Isolates with a greater percentage of identity than 98.7% [6] and only one closest match for the same identity characteristics are classified up to the species level.

To assess the reliability of a phylogenetic tree, MEGA provides the Bootstrap test. This test uses the bootstrap re-sampling strategy, so you need to enter the number of replicates. For a given data set, applicable tests and the phylogeny inference method are enabled. Neighbor joining has an additional test (Interior Branch), which requires the same input as bootstrap

Supplementary Table 3: Spearman correlation between soil physico-chemical parameters and total number of *Streptomyces* (TNS) in the three stations A, B and C.

	Variables	Value-r (Spearman)	Significance P-value
**1**	TNS VS Electrical conductivity	-0,800	Not significant
**2**	TNS VS pH	-0.200	Not significant
**3**	TNS VS Organic carbon	0.500	Not significant
**4**	TNS VS Organic matter	1.000**	P≤0.01
**5**	TNS VS Total nitrogen	1.000**	P≤0.01
**6**	TNS VS Clay texture	0.211	Not significant
**7**	TNS VS Sandy texture	0.500	Not significant
**8**	TNS VS Silty texture	0.400	Not significant
**9**	TNS VS Magnesium	0.400	Not significant
**10**	TNS VS Aluminum	0.500	Not significant
**11**	TNS VS Silicon	-0.500	Not significant
**12**	TNS VS Potassium	-0.500	Not significant
**13**	TNS VS Calcium	-0.400	Not significant
**14**	TNS VS Oxygen	0.400	Not significant
**15**	TNS VS Sulfur	-1.000**	P≤0.01
**16**	TNS VS Chlorine	-1.000**	P≤0.01
**17**	TNS VS Phosphorus	0.400	Not significant
**18**	TNS VS Iron	-0.500	Not significant
**19**	TNS VS Manganese	0.400	Not significant
**20**	TNS VS Cu	-0.500	Not significant
**21**	TNS VS Zn	0.800	Significant

(**): The correlation is significant at the 0.01 level, (TNS): Total number of *Streptomyces*

Supplementary Table 4: Resistance profile of multi-drug resistant (MDR) clinical bacteria.

Test strains	Resistance to	Sensitivity to	Intermediate to
**Clinical *Escherichia coli* 16D1150**	AML, AMC, CXM, CTX, AMP, P, FD, SXT, CIP, OFX, VA	CRO	CN, F
**Clinical *Proteus vulgaris* 16C1737**	F, AMC, AML, CXM, CTX, SXT, AMP, P, FD	CN, CRO, OFX,	CIP, AK
**Clinical *Neisseria gonrrhoae*16D1170**	P, AMP, AML, NA, FMQ, OFX, TE, SXT, FD,	AMC, CRO, AN, CN, CRO, E, F	CIP,
**Clinical *Staphylococcus aureus*18K1052**	P, AMP, AMX, AMC, CRO, CIP, NA, FMQ, OFX	KF, CXM, AN, GN, E, SXT, FD, IPM, F	-
**Clinical *Enterococcus faecalis* 18K1386**	P, AMP, KF, CXM, CRO, AN, GN, CIP, NA, FMQ, OFX, E,FD	AMX, AMC, SXT,F	IPM

AMC: Amoxicillin + Ac.clavulanic; P: penicillin G; AMP: Ampicillin; KF: Cefalotin; CRO: Ceftriaxone; AN: Amikacin; CN: Gentamicin; CIP: Ciprofloxacin; NA: Alidixicacid; FMQ: Flumequie; OFX: Ofolxacin; E: Erythromycin; TE: Tetracycline SXT: Trimethoprim+Sulfamide; FD: Fusidicacid; F: Nitrofurantne; CXM: Cefuroxin; VA: Vaomycin; CTX: Cefotaxime. IPM:Imipenem; CAZ: Ceftazidim; TM: Tobramycin; FOX: Cefoxitin; TIC: Ticarcillin

References

1. Bouaziz, S. *et al.* Antifungal activity of *Streptomyces* sp.14 strain isolated from Ouargla (Southeast of Algeria): identification, production and characterization of the active substance. *Int. J. Biosci.* **9**, 45–56 (2016).
2. Das, R., Romi, W., Das, R., Sharma, H. K. & Thakur, D. Antimicrobial potentiality of *Actinobacteria* isolated from two microbiologically unexplored forest ecosystems of Northeast India. *BMC Microbiol.* **18**, 1–16 (2018).
3. Thakur, D., Yadav, A., Gogoi, B. K. & Bora, T. C. Isolation and screening of *Streptomyces* in soil of protected forest areas from the states of Assam and Tripura, India, for antimicrobial metabolites. *J. Mycol. Med.* **17**, 242–249 (2007).
4. Lee, E. J., Hwang, K. Y., Lee, H. S. & Chung, N. Characterization of a new *Streptomyces* sp. a1022 as a potential biocontrol agent. *J. Appl. Biol. Chem.* **54**, 488–493 (2011).
5. Badji, B., Riba, A., Mathieu, F., Lebrihi, A. & Sabaou, N. Activité antifongique d’une souche d'*Actinomadura* d'origine saharienne sur divers champignons pathogènes et toxinogènes. *J. Mycol. Med.* **15**, 211–219 (2005).
6. Rossi-Tamisier, M., Benamar, S., Raoult, D. & Fournier, P. E. Cautionary tale of using 16s rRNA gene sequence similarity values in identification of human-associated bacterial species. *Int. J. Syst. Evol. Microbiol.* **65**, 1929–1934 (2015).
